# Mixture models reveal multiple positional bias types in RNA-Seq data and lead to accurate transcript concentration estimates

**DOI:** 10.1371/journal.pcbi.1005515

**Published:** 2017-05-15

**Authors:** Andreas Tuerk, Gregor Wiktorin, Serhat Güler

**Affiliations:** Lexogen GmbH, Vienna, Austria; University of Calgary Cumming School of Medicine, CANADA

## Abstract

Accuracy of transcript quantification with RNA-Seq is negatively affected by positional fragment bias. This article introduces Mix^2^ (rd. “mixquare”), a transcript quantification method which uses a mixture of probability distributions to model and thereby neutralize the effects of positional fragment bias. The parameters of Mix^2^ are trained by Expectation Maximization resulting in simultaneous transcript abundance and bias estimates. We compare Mix^2^ to Cufflinks, RSEM, eXpress and PennSeq; state-of-the-art quantification methods implementing some form of bias correction. On four synthetic biases we show that the accuracy of Mix^2^ overall exceeds the accuracy of the other methods and that its bias estimates converge to the correct solution. We further evaluate Mix^2^ on real RNA-Seq data from the Microarray and Sequencing Quality Control (MAQC, SEQC) Consortia. On MAQC data, Mix^2^ achieves improved correlation to qPCR measurements with a relative increase in R^2^ between 4% and 50%. Mix^2^ also yields repeatable concentration estimates across technical replicates with a relative increase in R^2^ between 8% and 47% and reduced standard deviation across the full concentration range. We further observe more accurate detection of differential expression with a relative increase in true positives between 74% and 378% for 5% false positives. In addition, Mix^2^ reveals 5 dominant biases in MAQC data deviating from the common assumption of a uniform fragment distribution. On SEQC data, Mix^2^ yields higher consistency between measured and predicted concentration ratios. A relative error of 20% or less is obtained for 51% of transcripts by Mix^2^, 40% of transcripts by Cufflinks and RSEM and 30% by eXpress. Titration order consistency is correct for 47% of transcripts for Mix^2^, 41% for Cufflinks and RSEM and 34% for eXpress. We, further, observe improved repeatability across laboratory sites with a relative increase in R^2^ between 8% and 44% and reduced standard deviation.

## Author summary

RNA-Seq is a powerful tool for detecting and quantifying genes and gene isoforms. However, accurate quantification in genomic loci with multiple isoforms has proven difficult. This is due to the fact that the transcript generating an RNA-Seq fragment cannot be identified if multiple transcripts share the fragment sequence. Due to this ambiguity, transcript concentration is usually determined in a statistical framework by calculating the probability that a transcript generates an RNA-Seq fragment. Accurate estimation of this probability requires an accurate model of the transcript specific distributions of RNA-Seq fragments. However, fragment distributions in statistical models of RNA-Seq data are usually over-simplified. This article introduces the Mix^2^ (rd. “mixquare”) model which uses mixtures of probability distributions to model the transcript specific positional fragment distributions. Mix^2^ learns the mixture weights and approximates therefore the fragment bias in RNA-Seq data. We compare Mix^2^ on artificial and real RNA-Seq data to four state-of-the-art quantification methods. Our experiments show that Mix^2^ yields more accurate and repeatable quantification estimates and that it leads to more accurate detection of differential expression. We further show that the biases detected by Mix^2^ contradict the common assumption of a uniform fragment distribution.

This is a *PLOS Computational Biology* Methods paper.

## Introduction

RNA-Seq has established itself as a popular alternative to microarrays for the quantification of RNA transcripts. In contrast to microarrays, which measure the quantity of an RNA transcript by hybridization to a transcript specific oligonucleotide, RNA-Seq generates cDNA for fragments of the RNA transcript, which are sequenced by a next generation (NGS) sequencer. One advantage of RNA-Seq over microarrays is that it does not require prior knowledge of the nucleotide sequence of the RNA transcript, which is needed to produce a transcript specific hybridization probe, and that it can therefore detect and quantify novel RNA transcripts. In addition, quantification by RNA-Seq covers a wider dynamic range since microarrays suffer from signal saturation resulting in the truncation of abundance estimates for highly abundant transcripts [1].

Despite these advantages, obtaining accurate transcript quantification measurements from RNA-Seq has proven difficult. One of the main reasons for the inaccuracy is the failure of the statistical models used in the derivation of the measurements to properly represent biases inherent in RNA-Seq data. The statistical model of the original version of Cufflinks [2], for instance, assumes that the cDNA fragments generated by RNA-Seq are uniformly distributed along the transcripts. In reality, however, this assumption is rarely fulfilled and quantification measurements by this version of Cufflinks are therefore often inaccurate.

One type of bias affecting transcript quantification from RNA-Seq data is the result of a preference of the fragmentation, i.e. the process that generates cDNA fragments from RNA transcripts, to produce fragments at certain positions within the transcript, e.g. at the start and/or at the end of the transcript [3]. Hence, this type of bias is referred to as positional bias [4]. Positional bias can also be caused by a bias in the RNA itself, for instance, due to RNA degradation which results in a shortening of the RNA. Another kind of bias in RNA-Seq is introduced during ligation, amplification and NGS sequencing [5]. This bias is correlated to the RNA sequence of a transcript and is therefore called sequence specific bias [4]. The present article focuses on the first type of bias, i.e. the positional bias, and develops a model, Mix^2^ (rd. “mixquare”), which learns the positional bias in RNA-Seq data. In our experiments we compare Mix^2^ to Cufflinks [2, 4], eXpress [6], RSEM [7] and PennSeq [8] both on synthetic data and on real RNA-Seq data [9] generated from the Universal Human Reference (UHR) and Human Brain (HBR) samples of the Microarray Quality Control (MAQC) experiment [10].

The inclusion of bias models into the statistical models of RNA-Seq data has been investigated before. In [11] a model is proposed to account for the variability in read counts depending on the sequence surrounding the start of a fragment. The intention is similar to that of the fragment specific bias model [4], which has been implemented as an extension to Cufflinks [2]. In addition, [4] includes a non-parametric positional bias model, which can theoretically be trained with the EM algorithm. However, due to the large number of variables, this is only feasible for few transcript length dependent classes, for which statistics are collected in a small number of positional bins. As a result, [4] implements a positional bias model depending exclusively on the length of a transcript. Similar to [4] the generative model of RSEM [7, 12] uses a hidden variable for the positional bias, where the latter is estimated from the global bias observed in the complete RNA-Seq data set. Also in RSEM, therefore, does the positional bias model depend exclusively on the transcript length. The generative model of eXpress [6] differs from Cufflinks mainly in the order of fragment-length selection and implements an online rather than a batch EM algorithm. The implementation described in [6] is further restricted to a sequence specific bias, with a uniform positional bias similar to Cufflinks. The model developed in PennSeq [8] is again non-parametric and the large number of variables makes its training computationally prohibitive. For this reason, the bias model of PennSeq [8] is not included in the parameter update but is approximated by the overall bias in a gene locus and by the transcript specific reads. The method described in [13] is a model for gene read counts, which models bias by exon specific weights, which are estimated both for the complete data set and for individual genes. In [14] the authors focus on RNA-Seq data with 5’ bias which is the result of RNA degradation and use an exponential model for the fragment distributions. The model proposed in [15] is, again, a model for the read counts of a gene. Here the read counts are modelled by a quasi-multinomial distribution with a parameter that can be adapted to account for over and under dispersion.

Mix^2^ is, similar to [2, 4, 6–8, 12], a generative model for the probability of a fragment in an RNA-Seq data set. In comparison, however, the model for the positional fragment bias in Mix^2^ is parametric. This considerably simplifies its implementation removing the need for any restrictions of the non-parametric methods. At the same time, the model of the positional fragment bias in Mix^2^ is very versatile since mixtures of probability distributions can approximate distributions of arbitrary complexity. Section (Materials and methods) develops the theory of Mix^2^ in greater detail and shows how its parameters can be updated with the EM algorithm leading to simultaneous estimates for transcript abundances and transcript specific positional fragment biases. Section (Experiments on artificial data) optimizes the number of mixture components of the Mix^2^ model and compares it with Cufflinks, RSEM and eXpress on artificial data sets. Sections (Experiments on the Microarray Quality Control (MAQC) data) and (Experiments on the Sequencing Quality Control (SEQC) data) on the other hand, discuss experiments on two publicly available real RNA-Seq data sets with Mix^2^, RSEM, eXpress, Cufflinks and PennSeq. These experiments show that, in comparison to the other methods, Mix^2^ leads to better correlation between estimated and measured transcript concentrations, correct recovery of mixing ratios and yields consistent titration orders. In addition, we show that the Mix^2^ concentration estimates are repeatable across lanes and laboratory sites and lead to a more accurate detection of differentially expressed transcripts. In addition, Section (Types of bias in the MAQC data) shows that Mix^2^ can be used as an explorative tool to detect positional biases present in RNA-Seq data. Mix^2^ has been implemented as an Octave script with readable code and as a closed source C++ implementation. We used the latter for the majority of our experiments. Both versions can be downloaded from https://www.lexogen.com/mix-square-scientific-license. While the C++ version is considerably faster, we show at the end of Section (Experiments on artificial data) and in Fig E in S2 Appendix that quantification results for both implementations are virtually identical. Hence, either version can be used to evaluate the accuracy of Mix^2^.

## Results

### The Mix^2^ RNA-Seq model: A mixture of mixtures

An essential part of Next Generation Sequencing (NGS) is the library preparation. This process takes an RNA sample and produces a library of short cDNA fragments, each corresponding to a section of an RNA transcript. The cDNA fragments are sequenced by an NGS sequencer resulting in single or paired end reads which are mapped to a reference genome. Hence, the probability *p*(*r*) of a fragment *r* can be interpreted as the probability of its genomic coordinates. In a genomic locus the probability *p*(*r*) is the superposition of the fragment distributions *p*(*r*|*t* = *i*) for the *N* transcripts in the locus, i.e.

$$\begin{array}{c} p\left(r\right)=\sum_{i=1}^{N}\alpha_{i}p\left(r|t=i\right) \end{array}$$ (1)

where *α*_*i*_ is the relative abundance of transcript *t* = *i*, i.e. the probability that transcript *t* = *i* generates any fragment, and *p*(*r*|*t* = *i*) is the probability that transcript *t* = *i* generates fragment *r*. Hence *p*(*r*|*t* = *i*) models the transcript specific fragment bias. An estimate for the concentration of transcript *t* = *i* is obtained by normalizing the relative abundance *α*_*i*_, yielding the RPKM [16] or FPKM values [2].

Mix^2^ uses a mixture model for *p*(*r*|*t* = *i*), i.e.

$$\begin{array}{c} p\left(r|t=i\right)=\sum_{j=1}^{M}\beta_{ij}p\left(r|t=i,b=j\right) \end{array}$$ (2)

where *p*(*r*|*t* = *i*, *b* = *j*) are the *M* components of the mixture and the *β*_*ij*_ are the non-negative component weights. Hence, *p*(*r*|*t* = *i*, *b* = *j*) is a probability distribution over *r* and

$$\begin{array}{c} \sum_{j=1}^{M}\beta_{ij}=1. \end{array}$$ (3)

Since *p*(*r*) is itself a mixture of the *p*(*r*|*t* = *i*) with weights *α*_*i*_, this implies that *p*(*r*) is a mixture of mixtures motivating the name of Mix^2^. For the *p*(*r*|*t* = *i*, *b* = *j*) we use Gaussians placed equidistantly along the transcript *t* = *i* (Materials and methods ).

**Fig 1:**
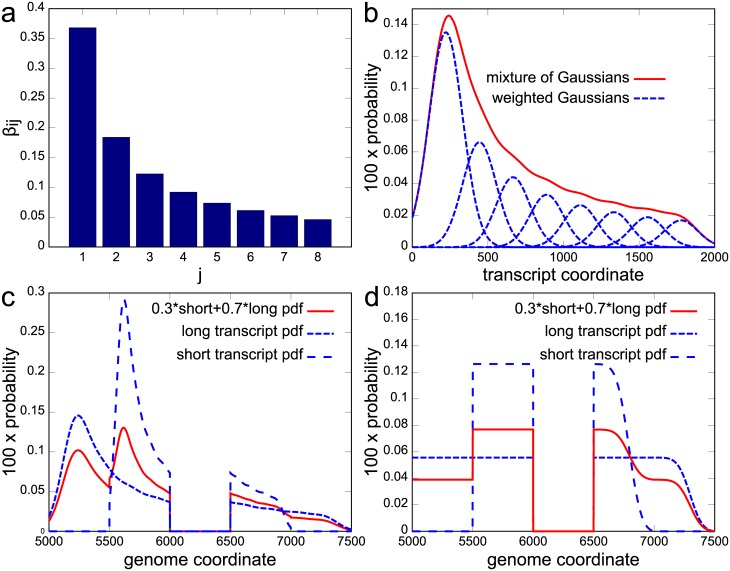
Fragment start distributions modelled by Mix^2^ model and Cufflinks. **(a)** Set of eight *β*_*ij*_. **(b)** Dashed curves: *β*_*ij*_*p*(*r*|*t* = *i*, *b* = *j*). The *p*(*r*|*t* = *i*, *b* = *j*) are Gaussians equidistantly distributed along a 2000 bp transcript. Solid curve: *p*(*r*|*t* = *i*). **(a)** and **(b)** in transcript coordinates. **(c)**, **(d)** Fragment distributions in locus with two transcripts, 1000 bp and 2000 bp long, sharing one junction. Long and short transcripts start 5000 bp and 5500 bp from beginning of locus contig. Junction starts at 6000 bp, extends to 6499 bp. Dashed curves: *p*(*r*|*t* = *i*) for Mix^2^ model **(c)**, Cufflinks **(d)**, long and short transcript. Solid curve: *p*(*r*) for Mix^2^ model **(c)**, Cufflinks **(d)**. **(c)** and **(d)** in genome coordinates.

An example for such a Mix^2^ model can be found in Fig 1. Fig 1(a) shows the mixture weights *β*_*ij*_ whereas Fig 1(b) shows the weighted Gaussians, *β*_*ij*_*p*(*r*|*t* = *i*, *b* = *j*), and the sum of the weighted Gaussians, *p*(*r*|*t* = *i*). The distributions in Fig 1(b) are given in transcript coordinates for a transcript of 2000 bp length, while the longer dashed curve in Fig 1(c) shows *p*(*r*|*t* = *i*) in genome coordinates. The locus in Fig 1(c) contains two transcripts which share a common junction. The shorter of the transcripts in Fig 1(c) has the same set of *β*_*ij*_ as in Fig 1(a) but is only 1000 bp long. The relative abundances of the long and short transcript are 0.7 and 0.3, respectively, which results in the overall distribution *p*(*r*) given by the solid curve in Fig 1(c). In comparison, Cufflinks [2] can, for this locus, only model fragment start distributions *p*(*r*|*t* = *i*) as visualized by the dashed curves in Fig 1(d) and is therefore inappropriate for 5’ biases as the one in Fig 1(c).

We use the Expectation Maximization (EM) algorithm (Materials and methods) to learn the parameters of Mix^2^ from RNA-Seq data. This results in simultaneous estimates of the transcript abundances *α*_*i*_ and the mixture weights *β*_*ij*_, hence the transcript specific fragment distributions *p*(*r*|*t* = *i*). Mix^2^ is identifiable in most cases and can otherwise be easily made identifiable (see Section 1.2 in S1 Appendix). It can therefore always be ensured that the EM algorithm converges to the unique maximum likelihood solution. It should be pointed out that we did not check for identifiability in our implementation and, like Cufflinks, returned the maximum likelihood solution produced by our method.

**Fig 2:**
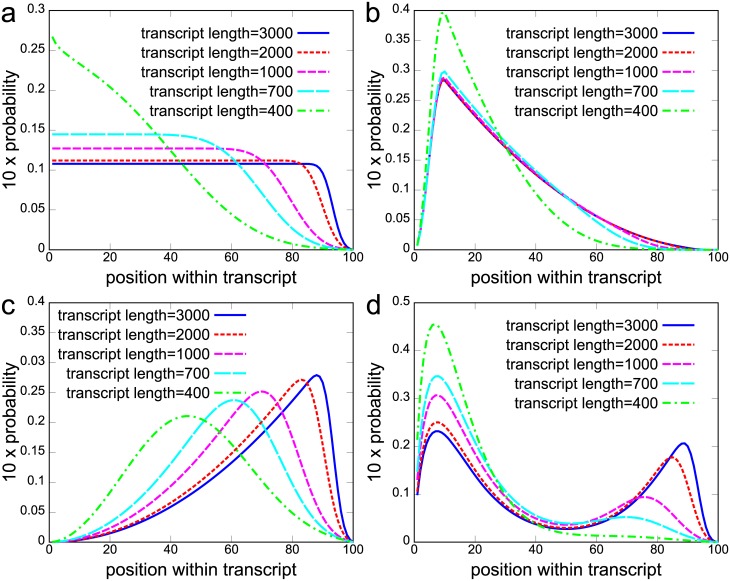
Transcript length dependent fragment start distributions in artificial data. X-axis is position within transcript in percent. Distributions are derived from an initial distribution, which is scaled to the transcript length. Subsequent multiplication with fragment start distribution of Cufflinks (a) and renormalization. (a) Distributions for uniform initial distribution. Corresponds to Cufflinks model for Gaussian fragment length distribution with mean 200 and standard deviation 80. Bias in (a) is referred to as Cufflinks bias. Distributions derived from initial distribution with 5’ bias (b), 3’ bias (c) and 5’+3’ bias (d).

The mixture weights *β*_*ij*_ determine the shape of the fragment distribution of transcript *t* = *i*. Thus, if transcripts have a similar distribution they should share the same *β*_*ij*_. This results in their fragment distributions being identical and reduces the number of parameters in Mix^2^ making it less prone to over-fitting. Consider, for instance, the fragment start distribution of the Cufflinks model in Fig 2(a). Here the distributions are similar for transcripts with 2000 bp and 3000 bp length and for transcripts with 700 bp and 1000 bp length. In this situation therefore, these four transcripts can be separated into two groups where the transcripts within each group share the same mixture weights *β*_*ij*_. In general, this leads to the scenario where each transcript *t* = *i* has an associated group *g* = *k* and the distributions *p*(*r*|*t* = *i*) of transcripts within this group share the same *β*_*ij*_. Multiple factors might influence the similarity of fragment start distributions. Here, we investigate gene membership and, as the bias correction methods in [4, 12], transcript length. The rationale for choosing these two properties is that even if fragments are uniformly distributed immediately after fragmentation, fragment size selection introduces the transcript length dependent bias in Fig 2(a). On the other hand, transcripts belonging to the same gene can share a substantial part of their sequence and exhibit therefore potentially similar fragmentation properties. In the following, we refer to tying between all transcripts within a gene as global tying and to tying between all transcripts within a gene and the same length range as group tying (Materials and methods). Hence, Fig 1(c) shows an example for a Mix^2^ model with global tying since both transcripts share the same set of weights *β*_*ij*_.

In our experiments we do not treat multi-mapping and uniquely mapping reads differently and instead consider each read mapping to be associated with a unique fragment. Multi-mapping reads and sequence specific bias can, however, be integrated into Mix^2^ as shown in Section 1.1 in S1 Appendix.

### Experiments on artificial data

This section pursues two goals. First, we find a sensible number of mixture components for each of the variants of Mix^2^. Second, we investigate the performance of Mix^2^ under conditions favoring other quantification methods. The number of mixture components derived in this section is used as a default by Mix^2^. While this might be suboptimal in some cases it is a necessary compromise since reference measurements of isoform concentrations on which to optimize the number of mixture components are usually not available in RNA-Seq data.

**Fig 3:**
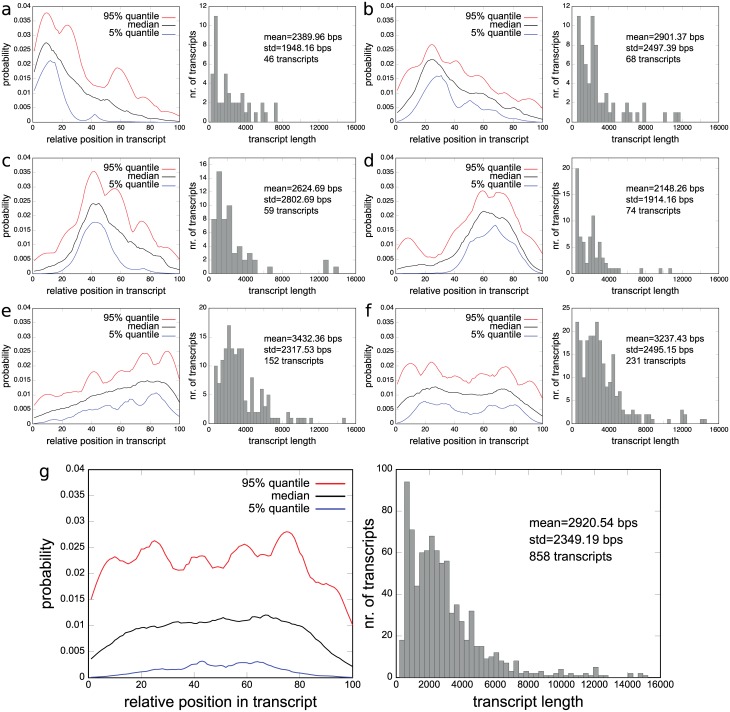
Types of biases detected in lane SRR037445 of UHR in MAQC data set and their transcript length distributions. (a) to (f) 6 most prominent biases which account for 73.43% of transcripts. Bias on the left, transcript length distribution on the right. (g) Bias and transcript length distribution of complete, unclustered set of transcripts.

In our first experiments we studied the 4 transcript length dependent biases in Fig 2. These resemble the biases we detected in our experiments on real RNA-Seq datasets MAQC and SEQC. The most dominant biases in the MAQC data are visualized in Fig 3. The 5’ biased fragment distributions in Fig 3(a) and 3(b), for instance, resemble the biases in Fig 2(b) and the biases for short transcripts in Fig 2(a). Similarly, the fragment distributions concentrated on the 3’ side in Fig 3(e) resemble the biases in Fig 2(c). While the biases in Fig 3 do not depend on transcript length as strongly as the biases in our artificial data, we see with the exception of Fig 3(d) an increase in the average transcript length with increasing 3’ bias. Overall, around 20.16% of transcripts have a 5’ bias in the MAQC data, 26.34% have a 3’ bias and 26.92% a uniform fragment distribution. Hence, the biases in our artificial data represent a sensible starting point for evaluating the accuracy of quantification methods under real-life conditions. Fig 2 also illustrates how an incorrect choice of bias model can affect the accuracy of quantification estimates. If the 5’ bias in Fig 2(b) is observed for a transcript of length 3000 bp but the bias model in Fig 2(a) is used for quantification then the incorrect model will try to explain the bias in Fig 2(b) with a shorter transcript, thus potentially leading to an overestimate of the concentration of the short transcript.

**Fig 4:**
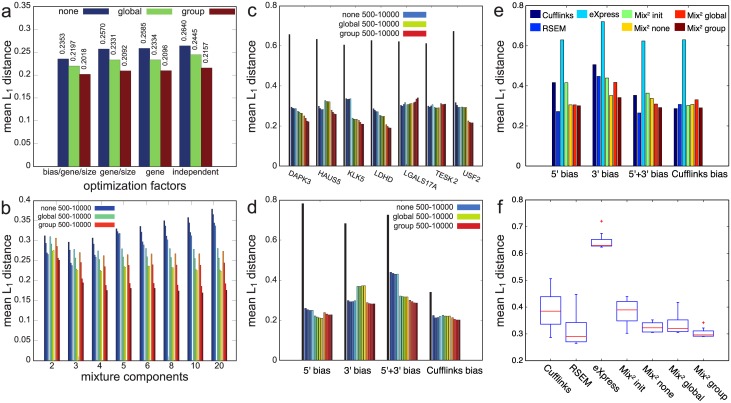
Average L_1_ distance on artificial data between true and estimated abundances and fragment start distributions. (a) Optimization of number of mixture components with regards to factors on x-axis (independent = no factors). Groups of 3 blocks give average L_1_ distance between abundances for Mix^2^ without tying (none), with global and group tying. (b) Dependence of quantification accuracy on number of mixture components and sample size. The three groups of four bars for each mixture number give L_1_ distance between abundances for Mix^2^ without tying, with global and group tying. Four bars within each group correspond to 500, 1000, 5000 and 10000 fragments. (c) and (d) L_1_ distance of fragment start distributions for Mix^2^ on 7 test genes and 4 biases. Tall bars indicate L_1_ distance for initial fragment start distributions of Mix^2^ before estimation of mixture weights *β*_*ij*_. First four of remaining bars represent Mix^2^ without tying, second and third group of four bars Mix^2^ with global and group tying. (e) and (f) Mean L_1_ distance between estimated and correct relative abundances for Cufflinks, RSEM, eXpress and Mix^2^ for 4 biases on full transcriptome and total of 50 mio read pairs. Experiments include results for Mix^2^ with initial fragment start distributions before estimation of mixture weights *β*_*ij*_ (Mix^2^ init). (f) boxplots of mean L_1_ distances in (e) for all methods.

In our experiments we used a set of 7 test genes from the GRCh37/hg19 Ensembl annotation v75 containing between 4 and 15 transcripts as well as the main variants of differential splicing, see Table A in S2 Appendix. While this set of genes might seem small, considering all parameter combinations resulted in 538k experiments. The difference between true and estimated relative isoform abundances is measured with the L_1_ distance, which is the sum of the absolute differences between true and estimated relative isoform abundances. This value lies between 0 and 2. In our experiments in Fig 4(a) we addressed the question if the optimal number of mixture components for Mix^2^ depends on the gene, the number of fragments in the gene or the fragment bias. In the first three bars of Fig 4(a) we optimized the number of mixture components for each combination of bias, gene and sample size separately and plotted the average L_1_ distance over all experiments for the three types of Mix^2^. In the second group of bars we optimized the number of mixture components for each combination of gene and sample size ignoring the bias, while in the third group we optimized for each gene ignoring bias and sample size. Finally, for the last three bars in Fig 4(a) we selected a single number of mixture components for all experiments independent of gene, bias and sample size. Overall, Fig 4(a) shows that little is gained by optimizing for each gene, sample size and bias separately. The number of mixture components of Mix^2^ can be chosen independent of these factors. Fig 4(b) shows the influence of the number of mixture components on the quantification accuracy of Mix^2^. For each number of mixture components on the x-axis the average L_1_ distance is given for different sample sizes. Each group of 4 blocks contains results for 500, 1000, 5000 and 10000 fragments, where the different groups show results for the different types of Mix^2^. Fig 4(b) implies that the minimal L_1_ distance for Mix^2^ without tying is obtained for 3 mixture components, while for Mix^2^ with group and global tying the minimum is obtained with any number between 4 and 10. In all our subsequent experiments we therefore chose 3 mixture components for Mix^2^ without tying and 4 mixture components for Mix^2^ with global and group tying.

We further studied the convergence of the fragment start distributions *p*(*r*|*t* = *i*) estimated by Mix^2^ to the correct start distributions from which the data was sampled. For this purpose, we calculated the average L_1_ distance between estimated and true distribution for the transcripts within a gene weighted by the estimated relative abundance of the transcript. Fig 4(c) and 4(d) visualize the difference between the L_1_ distance before (tall bars) and after estimation of the fragment start distributions. The initial fragment start distribution in Mix^2^ is close to uniform and independent of tying structure and read depth (Materials and methods). Fig 4(c) shows that estimates of the fragment start distributions in Mix^2^ converge to the correct solution since the L_1_ distance for the 7 test genes decreases during the course of the parameter estimation by between 50% and 65%. Mix^2^ with group tying and a read depth of 10000 fragments achieves in many cases the smallest final L_1_ distance. In Fig 4(d) the relative decrease in L_1_ distance is considerably smaller for the Cufflinks bias as the initial fragment start distribution in Mix^2^ is already close to the uniform Cufflinks distribution. Overall, the final L_1_ distance is similar for all 4 biases.

Next, we compared the accuracy of Mix^2^, Cufflinks, RSEM, and eXpress on the full transcriptome. We generated an exponential transcript expression profile from an exponential gene expression profile by FluxSimulator [17], as shown in Fig F and Fig G in S2 Appendix, and divided the gene reads between the gene transcripts according to relative abundances drawn from a uniform Dirichlet distribution. In total we generated around 50 mio 100 bps read pairs for each bias in Fig 2, which we aligned with Tophat2. Data of this kind correspond to the positional bias models in Cufflinks [4] and RSEM which are derived by scaling a single prototype distribution to the transcript length. Our experiments therefore show whether Mix^2^ can learn data structures hard-coded into the statistical models of Cufflinks and RSEM. Fig 4(e) shows that on 5’ and 5’+3’ bias RSEM slightly outperforms Mix^2^ with group tying, while for 3’ bias Mix^2^ yields the best results. On Cufflinks bias all methods apart from eXpress perform similarly. Fig 4(e) further contains results for Mix^2^ with initial fragment start distributions before estimation of mixture weights *β*_*ij*_ (Mix^2^ init). This shows that adapting the mixture weights improves relative abundance estimates. For Cufflinks bias this improvement is minor since the initial fragment start distributions in Mix^2^ are already close to the correct solution. The bad performance of eXpress is likely due to the fact that it models only sequence-specific and not positional bias. Fig 4(f) shows that Mix^2^ without any tying and with group tying perform consistently across biases and slightly better than RSEM with mean L_1_ distance of 0.36 versus 0.39. For Mix^2^ without parameter tying this is remarkable, as its statistical model is completely assumption free with regards to the nature of fragment bias. We also performed experiments for a smaller read-depth of 5 mio read pairs. These experiments which are summarized in Fig H in S2 Appendix show an increase of L_1_ distance for all methods but otherwise a similar trend as the experiments with 50 mio read pairs.

Finally, we compared the accuracy of the Octave and C++ version of our code, since on larger data sets we used, for efficiency reasons, the closed source C++ version. We found on our 7 test genes that the median L_1_ distance between the relative abundances estimated by the two versions was between 0.01 and 0.004 for 500 and 10k reads per gene. The boxplots for these experiments can be found in Fig E in S2 Appendix. Due to the small difference, either version of our code can be used to evaluate the accuracy of Mix^2^.

### Experiments on the Microarray Quality Control (MAQC) data

This section compares the accuracy of Mix^2^, Cufflinks [4, 18], PennSeq [8], RSEM [7] and eXpress [6] on two publicly available real RNA-Seq data sets generated from the Universal Human Reference (UHR) RNA and human brain (HBR) RNA of the Microarray Quality Control (MAQC) data [10] (Materials and methods). Both Cufflinks and RSEM were run with bias correction. In accordance with our experiments on artificial data we used three mixture components for Mix^2^ with no tying and 4 mixture components for Mix^2^ with global and group tying. We treat the qPCR measurements as a reference and compare them to the FPKM values generated by the quantification methods. The qPCR concentrations in the MAQC data are unevenly distributed spanning several orders of magnitude. It is therefore customary to compress the concentrations by taking the logarithm. This achieves a more even distribution but leads, on the other hand, to outliers for FPKM values close to zero whose logarithm approaches minus infinity. In order to reduce the influence of these outliers on the quality metrics for the quantification methods it is necessary to either remove transcripts with small FPKM values or to truncate the logarithm of their FPKM values. We chose the latter strategy since the former ignores the fact that some quantification methods fail to detect highly abundant transcripts and furthermore leads to test sets varying significantly in size reducing the comparability of the quality metrics. In our experiments, the logarithm of an FPKM value was truncated if it was below the first quartile minus 1.5 times the interquartile range of the logarithms of the method’s FPKM values on the test set. This threshold, which was between 0.01 and 0.001 in our experiments, is the point below which values are often considered to be outliers in boxplots. We use the logarithm to the basis of 10 in our experiments.

#### Accuracy of quantification estimates

In order to compare the accuracy of the quantification estimates of the different methods we measure the correlation between qPCR and FPKM values. In addition, we study the transcripts with truncated FPKM values. These transcripts can be considered not detected by the quantification method and their number should therefore ideally be small. We also calculate the average of the logarithms of their qPCR values. This should ideally also be a small number as it is more acceptable to not detect transcripts with low rather than high abundance.

**Fig 5:**
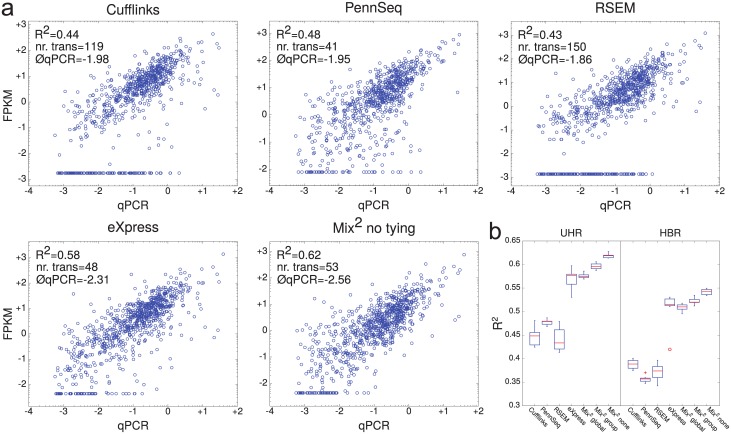
Correlation between qPCR and FPKM. (a) Typical lane on UHR for each of the 5 methods. The R^2^ value of each lane corresponds to the median of the R^2^ values over all 7 lanes of the respective method. The straight line of points at the bottom of the graphs represents the truncated FPKM values, whose corresponding transcripts can be considered not detected. The number of not detected transcripts and the average logarithm of their qPCR values are also given in the graphs. (b) Boxplots of R^2^ value over all 7 lanes of UHR and HBR.

Fig 5(a) shows that for typical lanes on UHR PennSeq, eXpress and Mix^2^ have a similar number of not detected transcripts, i.e. 41, 48 and 53, while this number is considerably higher for Cufflinks and RSEM, i.e. 119 and 150. The average number of the qPCR values of not detected transcripts is similar for Cufflinks, PennSeq and RSEM, i.e. -1.98, -1.95 and -1.86 and for eXpress and Mix^2^ with -2.31 and -2.56. Thus the latter two methods fail to detect only transcripts with low abundance while the former three also fail to detect highly abundant ones.

The boxplots in Fig 5(b) show that in comparison to the artificial data Mix^2^ without tying outperforms Mix^2^ with group and global tying. The R^2^ values of Mix^2^ are slightly higher than for eXpress on UHR and HBR with medians 0.62 and 0.54 versus 0.58 and 0.51. It should be noted, however, that on one of the 7 lanes of HBR eXpress produced an outlier yielding substantially worse correlation than on the other 6 lanes with an R^2^ value of 0.4. This outlier is indicated by the circle in the boxplots for eXpress. Overall, the correlation between qPCR and FPKM values suggests that Mix^2^ has a slight advantage over eXpress in terms of R^2^ value and fails to detect slightly fewer transcripts which also have a lower abundance. Both eXpress and Mix^2^ outperform the remaining 3 methods.

#### Repeatability of quantification estimates

**Fig 6:**
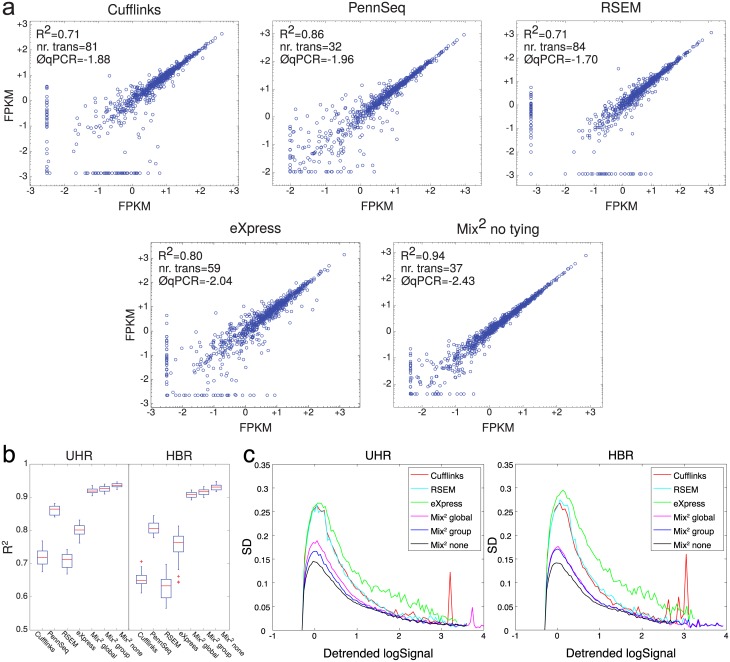
Repeatability of FPKM measurements for identical transcripts and samples in different lanes. (a) Typical lane pairs on UHR for each of the 5 methods. The R^2^ value of each lane pair corresponds to the median of the R^2^ values over all 21 lane pairs of the respective method. The straight lines of points at the bottom and the left side of the graphs represent transcripts detected in one lane but not the other. The number of these transcripts and the average logarithm of their qPCR values are also given in the graphs. (b) Boxplots of R^2^ value over all 21 lane pairs of UHR and HBR. (c) Variance based measure of repeatability. X-axis shows the average of the detrended log FPKM values. Y-axis shows the median standard deviation for all transcripts within one bin. X-axis was divided into 100 bins.

This section discusses the repeatability of quantification estimates for identical transcripts and samples on different lanes. Fig 6(a) shows that for the 5 typical lane pairs of UHR Mix^2^ without tying has the highest R^2^ value of 0.94. The number of transcripts which Mix^2^ does not detect in one of the lanes is slightly higher with 37 than for PennSeq with 32, but the average qPCR value of these transcripts is considerably lower with -2.43 than -1.96 for PennSeq. The other 3 methods perform noticeably worse than the Mix^2^ model.

Fig 6(b) shows that on the complete 21 lane pairs in both UHR and HBR the FPKM values of Mix^2^ are consistently better correlated and their correlation is less variable than those of the 4 other quantification methods. On HBR, for instance, the median of the R^2^ value for Mix^2^ is 0.93, whereas PennSeq achieves 0.88. In addition to the R^2^ value, we also evaluated the repeatability of quantification estimates in Fig 6(c) with the variance based measure from [19]. Since we performed evaluation on the complete transcriptome we had to exclude PennSeq since it failed to produce a result on the vast majority of the data. FPKM values where detrended such that all methods had the same median of log FPKM values on the house keeping genes in [20]. The x-axis in Fig 6(c) represents the average concentration over all 7 lanes while the y-axis represents the median standard deviation. Fig 6(c) shows therefore that over the complete concentration range Mix^2^ without tying achieves the best repeatability on both UHR and HBR followed by the variants with group and global tying. In contrast to the experiments in Fig 6(a) and 6(b), the variance based measure rates the repeatability of eXpress worse than that of RSEM and Cufflinks.

#### Accuracy of fold change estimates and the detection of differential expression

**Fig 7:**
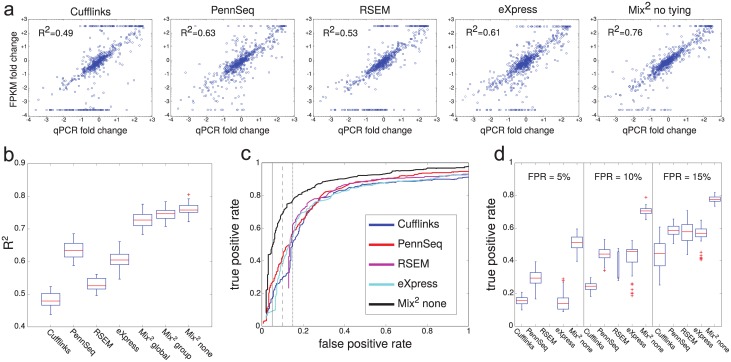
Correlation of qPCR and FPKM fold changes between UHR and HBR and accuracy of differential expression detection. (a) Typical lane pairs for each of the 5 methods. The R^2^ value of each lane pair corresponds to the median of the R^2^ values over all 49 lane pairs of the respective method. FPKM fold changes were truncated if they were larger or smaller than the maximal or minimal qPCR fold change. The straight lines of points at the top and bottom of the graphs represent truncated FPKM fold changes. (b) Boxplots of R^2^ value over all lane pairs in UHR and HBR. (c) ROC curves for classification experiment regarding differentially expressed transcripts in the lane pairs in (a). The vertical lines correspond to false positive rates of 5%, 10% and 15%. (d) Boxplots of true positive rates of classification experiment on all 49 lane pairs in UHR and HBR for false positive rates of 5%, 10% and 15%.

Fold changes of concentration measures between different samples are used in the analysis of differential expression both for microarrays [21] and RNA-Seq [2]. A high correlation between the qPCR and FPKM fold changes is therefore important to ensure accurate differential expression calls. Technical variability added by low correlation, on the other hand, leads to a loss of power of statistical tests for differential expression, such as [22] and [23]. This section analyses the correlation of FPKM and qPCR fold changes between UHR and HBR. For the 5 typical lane pairs of UHR and HBR in Fig 7(a) Cufflinks and RSEM produce a large number of high FPKM fold changes for transcripts with a small qPCR fold change. As a result, the R^2^ value of the correlation between FPKM and qPCR fold changes for Cufflinks and RSEM is smaller than for the other methods. The boxplots in Fig 7(b) show that the Mix^2^ model yields consistently higher correlation between FPKM and qPCR fold changes on all 49 lane pairs of UHR and HBR.

In order to determine the influence of the correlation of FPKM and qPCR fold changes between UHR and HBR on the detection of differential expression, a simple classification experiment was performed, similar to an experiment in [9]. Transcripts with a qPCR fold change above 2 were defined as differentially up-regulated while transcripts with a qPCR fold change below 0.5 were defined as differentially down-regulated. The remaining transcripts were defined as not differentially expressed. Subsequently all transcripts were classified according to their FPKM fold change. If the FPKM fold change was above a certain threshold the transcript was classified as up-regulated, whereas it was classified as down-regulated if its FPKM fold change was below the inverse of the threshold. The threshold varied between 1.1 and the maximal FPKM fold change for the method. For each threshold the true and false positive rate, with respect to the qPCR based definitions, were recorded. FPKM fold change outliers are acceptable in this experimental setup and were therefore not truncated as before. Fig 7(c) shows that the true positive rate in the classification experiments for the 5 lane pairs in Fig 7(a) is consistently higher for Mix^2^ than for the other methods. For false positive rates of 5%, 10% and 15% this difference is particularly strong as indicated by the vertical lines in Fig 7(c). Unlike the other methods, the ROC curve of RSEM does not reach the origin of Fig 7(c), which is due to the large number of transcripts having a small qPCR and an infinite FPKM fold change. The boxplots in Fig 7(d) show that Mix^2^ produces considerably higher true positive rates for false positive rates of 5%, 10% and 15% than the other 4 methods on the complete 49 lane pairs from UHR and HBR. For the false positive rate of 5%, for instance, the median of the true positive rates for Mix^2^ and PennSeq is 51% and 29%, respectively. For RSEM, in contrast, there is no lane pair for which the ROC curve obtains a false positive rate of 5%.

#### Resource usage

We performed experiments to determine the CPU usage and memory footprint of Mix^2^ and the other methods. PennSeq was excluded from these experiments because its unreliable implementation in PERL can only be regarded as a proof-of-concept. We ran Mix^2^, eXpress, RSEM and Cufflinks in single-core mode on the complete 7 lanes of UHR and HBR in parallel on an Intel Xeon E5620 with 2.4GHz and 8 physical cores. Only two instances of eXpress were run at the same time as eXpress uses a minimum of 3 threads. From these runs we derived the CPU usage for each lane as well as the maximal memory usage. The average numbers over all 7 lanes are collected in seconds and gigabytes in Table 1. This shows that Mix^2^ without tying is slightly faster than RSEM and both are considerably faster than eXpress and Cufflinks. In comparison to Cufflinks, Mix^2^ without tying is faster by a factor of 70 and 87 on UHR and HBR, respectively. In comparison to eXpress it is faster by a factor of 16 and 13. In terms of memory usage all methods are within reasonable bounds. RSEM uses the smallest amount, followed by Cufflinks and Mix^2^ which have roughly the same memory footprint, eXpress, on the other hand, uses about 4 times more memory than Mix^2^.

**Table 1:** Run-time and maximal memory usage on UHR and HBR.

	Mix^2^	Mix^2^ global	Mix^2^ group	eXpress	RSEM	Cufflinks
Run time						
mean UHR	407.39	427.01	461.21	7438.57	425.90	32522.00
std UHR	20.40	19.99	22.67	164.54	24.03	265.32
mean HBR	320.33	352.51	364.00	5032.15	367.28	32186.43
std HBR	24.19	28.48	26.75	413.70	20.52	378.58
Memory usage						
mean UHR	1.25	1.26	1.28	5.07	0.84	1.32
std UHR	0.05	0.05	0.05	0.00	0.03	0.02
mean HBR	1.02	1.02	1.04	5.07	0.79	1.22
std HBR	0.08	0.08	0.08	0.00	0.01	0.02

Time and memory usage are given in seconds and gigabytes, respectively. Values are averages over the 7 lanes in UHR and HBR.

#### Types of bias in the MAQC data

Mix^2^ simultaneously estimates relative abundances and transcript specific fragment distributions and can therefore be used to detect biases present in RNA-Seq data. Fig 3(a) to 3(f) visualize 6 bias types for a subset of transcripts in one lane of UHR, which were obtained by clustering the fragment start distributions learned by the Mix^2^ model. For this purpose the fragment start distributions of the 798 genes of the MAQC test set from one lane of UHR were selected, which were assigned a read count by Mix^2^ of at least 100. The minimum read count was set at 100 in order to avoid fragment start distributions which might be inaccurate due to a small amount of data. The 858 fragment start distributions, which satisfied these requirements were then length normalized and hierarchically clustered with UPGMA [24] using the L_1_ distance. The resulting tree was traversed from top to bottom and nodes were retained which had at least 5% or 43 of the 858 fragment start distributions. If a node was reduced in size at the next level of the tree without changing the overall shape of the distributions, the top node was chosen. This process resulted in the 6 clusters of fragment start distributions shown on the left side of Fig 3(a) to 3(f). Fig 3(g), on the other hand, shows the fragment start distribution for the complete unclustered data. The median in Fig 3(g) gives the false impression that the fragment start distributions can be modelled by a uniform distribution. Instead, the distributions separate into a class with 5’ bias, Fig 3(a) to 3(c), a class with 3’ bias, Fig 3(d) and 3(e), and a uniform class, Fig 3(f). The classes with 5’ or 3’ bias contain 46.50% of the complete fragment start distributions, while the uniform class contains only 26.92%. Overall, 73.43% of the distributions are contained in one of the classes in Fig 3(a) to 3(f). The remaining 26.57% of the distributions belong to classes each containing less than 5% of the distributions. Thus, biased fragment distributions represent the majority of the data. There exists, furthermore, no single bias type but multiple biases are observed.

### Experiments on the Sequencing Quality Control (SEQC) data

In order to evaluate the quantification methods on more recent data than MAQC, we also performed experiments on the SEQC data set [25]. The latter contains 100 bps paired-end RNA-Seq reads generated with Illumina HiSeq 2000 at 6 laboratory sites and from four different RNA samples. Samples A and B correspond to UHR and HBR from the MAQC data set. Sample C and D were created by mixing A and B in 3:1 and 1:3 ratios, respectively. This allows tests for titration order consistency and the correct recovery of mixing ratios. These tests are independent of a “gold standard” such as qPCR which is biased by its own technical limitations. In this section, therefore, we evaluate the accuracy of quantification with respect to the built-in ground truths of SEQC rather than with respect to qPCR measurements. For this purpose, we downloaded RNA-Seq data for samples A to D for laboratory site BGI. To study repeatability across sites we further downloaded RNA-Seq data for sample A and sites AGR, CNL and COH. We compare the three variants of Mix^2^ with Cufflinks, RSEM and eXpress. PennSeq had to be excluded as it failed to produce any output on the SEQC data. As before, we used GRCh37/hg19 and Ensembl annotation version 75 in our experiments and 3 mixture components for Mix^2^ without tying and 4 mixture components for Mix^2^ with global and group tying.

#### Accuracy of quantification estimates

Since we were not interested in inter-lane effects in this section, we pooled the data from all lanes in one flow-cell for each sample A to D for the BGI site. This resulted in 43 mio to 55 mio read pairs per sample. The reads were then mapped against the genome using tophat2 and against the transcriptome using bowtie2. Subsequently, we ran Cufflinks, RSEM, eXpress and Mix^2^ to obtain quantification estimates for samples A to D. As described in [25] we used the quantification estimates for each transcript in samples A and B together with the known mixing ratios to predict the ratio between transcript abundances in C and D. This predicted ratio was then compared to the ratio of the quantification estimates in samples C and D. A high similarity between prediction and measurement is desirable as it shows a method to correctly reflect the relative changes across different samples. In this respect, the experiments in this section answer a similar question as our experiments regarding FPKM and qPCR fold changes on the MAQC data.

**Fig 8:**
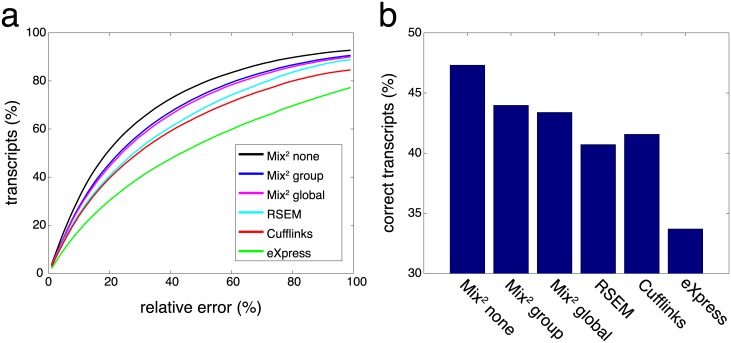
Evaluation on built-in ground truths of SEQC data set. (a) Relative error of predicted C/D ratio vs. measured C/D ratio. Y-axis shows the percentage of transcripts having a relative error less than value on x-axis. (b) Titration order consistency. Y-axis shows the percentage of transcripts with correct titration order.

Fig 8(a) shows on the x-axis the relative error of the predicted versus the measured C/D ratio. The y-axis of Fig 8 represents the percentage of transcripts with a relative error smaller than the value on the x-axis. Fig 8 shows therefore that for any given value on the x-axis the number of transcripts having a smaller relative error is higher for Mix^2^ than for the other methods. As before on MAQC, Mix^2^ without tying yields the best results followed by group and global tying.

Similar to the C/D ratio the titration order of all samples can be inferred from the titration order of A and B and the known mixing ratios. In particular, if quantification for a transcript yields *A* > *B*, then one should have *A* > *C* > *D* > *B* for this transcript, with reversed order if *B* > *A*. Fig 8(b) shows the number of transcripts with correct titration order. Again, Mix^2^ without tying yields the best results with about 47% of transcripts having a correct titration order in comparison to around 42% for Cufflinks and RSEM and 33% for eXpress.

#### Repeatability of quantification estimates

In the experiments in this section we evaluated repeatability across sites on sample A. As before, we used data from a single flow-cell. In contrast to the previous section, we performed quantification on individual lanes to capture lane-specific variations in repeatability. Lanes contained between 6 mio and 13 mio read pairs. We measured repeatability both with the R^2^ value and the variance based measure in [19]. To calculate the R^2^ value we truncated small FPKM values, as before, to a lane specific threshold. However, for Cufflinks we used the median of the thresholds for MAQC since Cufflinks produced large numbers of extremely small FPKM values resulting in a large negative threshold on the log scale. As a consequence, R^2^ values for Cufflinks were unusually small.

**Fig 9:**
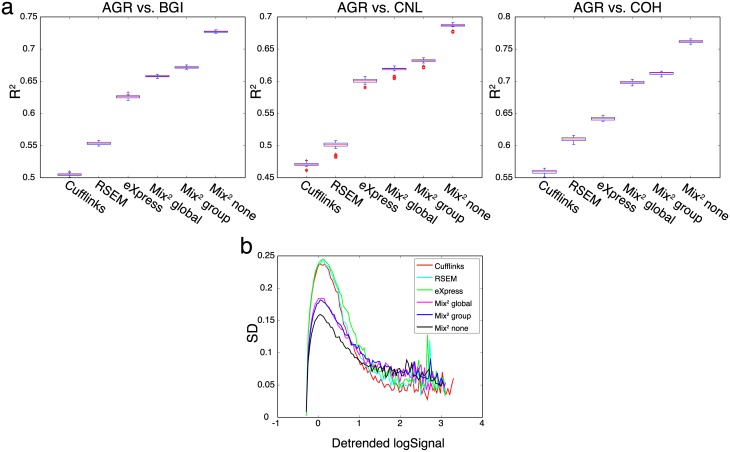
Repeatability across sites. (a) Boxplots of R^2^ values between lanes from two sites. All lanes from a single flow-cell are considered. Comparison between site AGR and sites BGI, CNL and COH. (b) Variance based measure of repeatability across all 4 sites and all lanes. Curves show the median standard deviation (y-axis) for transcripts with average detrended log signal (x-axis).

Fig 9(a) shows boxplots of the R^2^ values for comparisons between lanes from two sites. Here, all lanes from a single flow-cell are considered and site AGR is compared to sites BGI, CNL and COH. Not surprisingly, the R^2^ values are smaller for comparisons across sites than for the comparisons across lanes in the MAQC experiments in Fig 6. This is also reflected by the small variance of the boxplots in Fig 9(a). The boxplots for the remaining site comparisons can be found in Fig I in S2 Appendix and give a similar picture. Whereas Fig 9(a) is a pairwise comparison, Fig 9(b) shows the standard deviation over all lanes and sites versus the detrended log signal as defined in [19]. In the region where these curves can be estimated reliably, Mix^2^ without tying has the smallest standard deviation. This is consistent with the pairwise analysis in Fig 9(a) and Fig I in S2 Appendix.

#### Types of bias in the SEQC data

**Fig 10:**
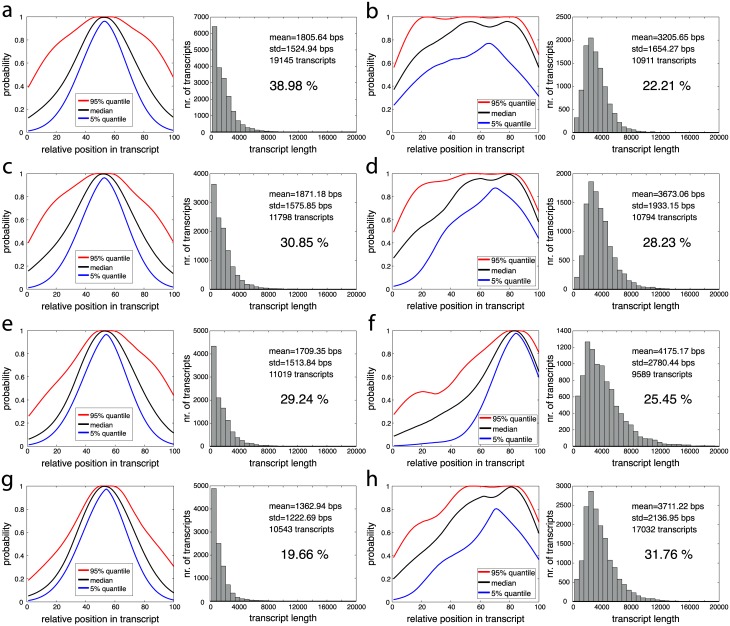
The two most prominent biases in sample A with corresponding transcript length histograms at site AGR (a) and (b), BGI (c) and (d), CNL (e) and (f) and at site COH (g) and (h). The histograms also show the site dependent percentage of transcripts contained in each cluster.

We further studied biases in the SEQC data by clustering the distribution of fragment start sites estimated by Mix^2^ with group tying and 3 mixture components. To obtain reliable estimates we pooled all lanes in a single flow-cell. In contrast to our experiments on the MAQC data, we used all the transcripts in the annotation. We only required that transcripts were between 200 bps and 20k bps in length and had a minimum of 100 fragments according to Mix^2^. This resulted in sets of between 38k and 54k transcripts. We used hierarchical pairwise average linkage clustering as implemented in [26] in conjunction with the L_1_ distance. Prior to clustering, distributions were length normalized and scaled such that their maximal value equaled one. The latter helps to decrease the L_1_ distance between visually similar distributions. Without scaling the algorithm produced a single cluster containing almost the entire data. Fig 10 shows the two most dominant bias types for the four evaluated sites on sample A. These bias types and their associated transcript length histograms are similar for all sites. One of the clusters consists of distributions concentrated around the middle of comparatively short transcripts while the other cluster contains slightly 3’ biased distributions for comparatively long transcripts. Also the remaining clusters are similar for all sites as can be seen from Fig J to Fig P in S2 Appendix. Biases are also similar across samples as can be seen for Fig M to Fig P in S2 Appendix which show clusters for samples A to D of BGI. While having very similar shapes and histograms, clusters sometimes appear to differ in their occupancy, as can be seen from the percentage of their distributions shown in Fig 10. However, these differences might be due to the clustering algorithm exaggerating small changes in the input data. Overall, the experiments in this section show strong similarities between biases at different sites and for different samples, which would be expected from applying the standard protocol of a library preparation.

## Discussion

This article introduced Mix^2^ which uses a mixture of probability distributions to model transcript specific positional fragment distributions in RNA-Seq data. Due to the flexibility of mixture models, Mix^2^ can adapt to multiple positional fragment biases of arbitrary complexity. The parameters of Mix^2^ are efficiently trained with the EM algorithm resulting in simultaneous estimates for fragment distributions and relative abundances. In addition, parameters of Mix^2^ can be tied between transcripts with similar fragment distribution leading to improved estimates of the relative abundances. Even though Mix^2^ accommodates a sequence specific bias, we currently implement only a model for positional bias. Sequence specific bias can, however, be a prominent feature in RNA-Seq data. In [11], for instance, it was found that a linear model on the sequences of 3 RNA-Seq data sets accounted for between 40% and 50% of the variance in sequence frequencies. Implementing a model for the sequence specific bias to Mix^2^ might therefore result in further improvements in the accuracy of transcript quantification.

Experiments were conducted on artificial data to determine the optimal number of mixture components of Mix^2^. These experiments showed that optimization can be performed independent of gene, bias and sample size and that the optimal number of mixture components is 3 for Mix^2^ without tying and any number between 4 and 10 for Mix^2^ with tying. These numbers are fairly small given the biases in Fig 2. In particular, it seems implausible that Mix^2^ with only 3 mixture components should be able to accurately model the 5’+3’ bias in Fig 2(d). This suggests that the potential of Mix^2^ has yet to be fully exploited. Our experiments, further, showed that the estimate of Mix^2^ for the transcript specific fragment bias converges to the correct distribution and that these estimates can therefore be used to detect positional bias present in RNA-Seq data.

Experiments were also performed on RNA-Seq data generated from Universal Human Reference (UHR) RNA and Human Brain (HBR) RNA for the Microarray and Sequencing Quality Control (MAQC, SEQC) data sets. On MAQC, we obtained improved correlation between qPCR measurements and quantification estimates with Mix^2^, while on the SEQC data set, Mix^2^ produced improved titration order consistency and recovery of mixing ratios. In addition, correlation and standard deviation of Mix^2^ quantification estimates were superior across lanes in MAQC and across laboratory sites in SEQC, implying reduced sensitivity to technical variance. Furthermore, correlation of qPCR and FPKM fold changes between UHR and HBR on MAQC were noticeably higher for the Mix^2^ model than for the other methods. We showed in a classification experiment that this leads to higher accuracy in the detection of differential expression. In general, quantification accuracy affects sensitivity and specificity of statistical tests for differential expression. Inconsistent quantification estimates, for instance, increase technical variability and lead to an increase in the variance of the data models underlying statistical tests. This, in turn, leads to a decrease of the chi-square distributed test statistic of the Wald test in DSS [27], DESeq2 [23], edgeR [22] and the score test in PoissonSeq [28] resulting in a loss of sensitivity. The same holds for the Wilcoxon test in SamSeq [32] where the test statistic moves closer to the mean of the test distribution under the 0-hypothesis. Likewise, conditional model probabilities in baySeq [29] and conditional probabilities in the exact tests of DESeq [30] and edgeR [31] become more uniform and therefore less distinctive between different conditions. The noise distributions in NOISeq [33, 34] and DEGSeq [36] become wider with increasing technical variability and fold change thresholds for the detection of differential expression increase, again reducing the sensitivity of these methods. For DEXUS [35] the variance of the major and minor conditions increases resulting in greater overlap. Since the prior of the model probabilities favors a single condition this makes it less likely that minor conditions and therefore differential expression will be detected. Overall, the aforementioned statistical tests benefit both in terms of sensitivity and selectivity from more accurate quantification estimates and we expect therefore to see improved differential expression calls for transcripts when using these tests in conjunction with Mix^2^.

In terms of resource usage, both RSEM and Mix^2^ take about the same time to process the 7 lanes of UHR and HBR in MAQC and are both faster than eXpress and Cufflinks. Memory usage of RSEM is slightly smaller than that of Mix^2^ and Cufflinks but memory consumption of all 4 methods is low given the specifications of current computing environments. In contrast to the experiments on artificial data, experiments on MAQC and SEQC showed a degradation in the performance of Mix^2^ when tying parameters. This cannot be attributed to a suboptimal choice of parameters in the transcript clustering procedure. Instead, it seems that positional fragment bias does not exclusively depend on gene membership and transcript length. This fact was also highlighted in our experiments on bias types in MAQC and SEQC. In these experiments we found dominant bias types by clustering fragment start distributions estimated by Mix^2^. On MAQC we obtained 6 clusters, containing 73.43% of the distributions, of which 5 clusters exhibited non-uniform distributions. The cluster with uniform distributions contained only 26.92%. On SEQC we also see the majority of distributions located in non-uniform clusters. In addition, clusters are similar across laboratory sites. Contrary to our experiments on artitifical data there is no obvious relationship between bias and transcript length, although correlations between the two do exist. For instance, transcripts whose fragment start distributions are 3’ biased or uniform tend to be longer, whereas 5’ biased transcripts tend to be shorter. A more detailed analysis of biases might reveal relations between positional bias and RNA sequences that will lead to a better tying strategy for the Mix^2^ model on real RNA-Seq data.

In summary, Mix^2^ can be used as an explorative tool to investigate the positional biases present in RNA-Seq data and thereby study the influence of library preparation, sequencing and data processing on the accuracy of transcript concentration estimates. In addition, and more importantly, our results show that Mix^2^ yields improved transcript concentration estimates for RNA-Seq data with higher repeatability for technical replicates and leads, furthermore, to improved accuracy in the detection of differential expression.

## Materials and methods

### Mixture components

We factorize *p*(*r*|*t* = *i*) as follows,

$$\begin{array}{c} p\left(r|t=i\right)=p\left(l\left(r\right)|t=i,s\left(r\right)\right)\sum_{j}\beta_{ij}p\left(s\left(r\right)|t=i,b=j\right) \end{array}$$ (4)

where *s*(*r*) and *l*(*r*) are the start and length of fragment *r* and *p*(*s*(*r*)|*t* = *i*, *b* = *j*) are Gaussians whose means *μ*_*ij*_ are placed equidistantly along the transcript. If one disregards the dependency on *s*(*r*) and *t* = *i* it is possible, similarly to Cufflinks, to estimate *p*(*l*(*r*)) from the data. For paired-end data, however, we always set *p*(*l*(*r*)) to a Gaussian with mean 200 and standard deviation 80, which is the default fragment length distribution for Cufflinks. The component weights *β*_*ij*_ are associated with equidistant positions within the transcript *t* = *i* and represent therefore the overall shape of *p*(*r*|*t* = *i*). In transcript coordinates the means *μ*_*ij*_ of the Gaussians are given by

$$\begin{array}{c} \mu_{ij}=j\cdot \frac{l(t=i)}{M}-\frac{l(t=i)}{\left(2M\right)} \end{array}$$ (5)

and their standard deviations are, independent of *j*, set to

$$\begin{array}{c} \sigma_{ij}=\frac{l(t=i)}{\left(2M\right)}. \end{array}$$ (6)

where *l*(*t* = *i*) is the length of transcript *t* = *i* and *M* is the number of mixture components. The Gaussians are, furthermore, normalized such that their sum over the possible fragment starts *s* = 1, …, *l*(*t*) equals one.

### Parameter estimation

The relative abundances *α*_*i*_ in Mix^2^ can be updated with the EM algorithm in the usual manner, as implemented, for instance, in Cufflinks [2]. This update formula is given in Section 1.1 in S1 Appendix. For the transcripts in group *g* = *k* the *β*_*ij*_ = *β*_*kj*_ can be updated with the EM algorithm as follows

$$\begin{array}{c} \beta_{kj}^{\left(n+1\right)}=\frac{\sum_{r}p^{\left(n\right)}\left(g=k,b=j|r\right)}{\sum_{r}p^{\left(n\right)}\left(g=k|r\right)} \end{array}$$ (7)

where

$$\begin{array}{c} p^{\left(n\right)}\left(g=k|r\right)=\sum_{i\in k}p^{\left(n\right)}\left(t=i|r\right) \end{array}$$ (8)

and

$$\begin{array}{c} p^{\left(n\right)}\left(g=k,b=j|r\right)=\sum_{i\in k}p^{\left(n\right)}\left(t=i,b=j|r\right) \end{array}$$ (9)

and the sums in Eqs (8) and (9) are extended over the transcripts *t* = *i* in group *g* = *k*. Here $\beta_{kj}^{\left(n+1\right)}$ and *p*^(*n*)^(·) are the mixture components and posterior probabilities after the *n*+1-th and after the *n*-th iteration, respectively. To calculate *p*^(0)^(·) it is necessary to initialize the model parameters, which we do as follows

$$\begin{array}{c} \alpha_{i}^{\left(0\right)}=\frac{1}{N},\beta_{kj}^{\left(0\right)}=\frac{1}{M}. \end{array}$$ (10)

where *N* is the number of isoforms in the gene locus and *M* is, again, the number of mixture components. Hence, our initial distributions *p*^(0)^(*r*|*t* = *i*) are close to uniform. EM iterations are repeated until changes in the model parameters or the overall likelihood of the model fall below a predefined threshold.

### Group tying

We initially place the transcripts of a gene into groups according to their length where the 7 transcript length boundaries are equidistantly distributed on the log scale between 300 and 5000. Subsequently, groups are merged until each group has at least 20 valid reads and there is at most one group containing a single transcript. Groups are merged according to their distance, which is calculated as follows

$$\begin{array}{c} \frac{|mean\left(g=k_{1}\right)-mean\left(g=k_{2}\right)|}{100-\min(mean\left(k_{1}\right),mean\left(k_{2}\right))} \end{array}$$ (11)

where *mean*(*g* = *k*) is the average length of transcripts in group *g* = *k*. The two closest groups are merged first.

### The artificial data set

For each of the 7 genes, 200 sets of abundances (*α*_1_, …, *α*_*N*_) were sampled uniformly, according to the Dirichlet distribution. Subsequently, for each of the 200 sets of abundances 500, 1000, 5000 and 10000 fragments were sampled from the superposition Eq (1), where the *p*(*r*|*t* = *i*) belong to one of the 4 bias models in Fig 2. These biases are referred to as Cufflinks bias (a), 5’ bias (b), 3’ bias (c) and 5’+3’ bias (d). The Cufflinks bias is the fragment start distribution of the Cufflinks model for a fragment length distribution with mean 200 bp and standard deviation 80 bp. The other biases in Fig 2 are derived by scaling an initial 5’, 3’ or 5’+3’ biased distribution to the length of the transcript. Subsequently, the scaled distribution is multiplied by the Cufflinks bias for the transcript length and renormalized. This explains why the 5’ tails of the biases in Fig 2 become increasingly heavy for shorter transcripts. Section 2 in S1 Appendix contains a brief discussion of how the Cufflinks bias is derived from the Cufflinks model and Fig A to Fig D in S2 Appendix show examples for the coverage resulting from sampling the biases in Fig 2. The fragment lengths *l*(*r*) were sampled from a Gaussian with mean 200 bp and standard deviation 80 bp and the resulting fragments were then converted into 50 bp paired-end reads and written to a SAM file [37]. Thus, 800 data sets were generated per gene and sample size or, equivalently, 1400 data sets per bias model and sample size resulting in a total of 22400 data sets. On each of these data sets Mix^2^ was run without tying as well as with group and global tying, where the number of mixture components ranged from 2 to 20. Hence a total of 537600 experiments were performed with Mix^2^ on these artificial data.

For the experiments on the complete transcriptome we used only genes with multiple transcripts since the estimated and true relative abundance on genes with a single transcript is always one and their L_1_ distance is therefore zero. Hence, accumulating the L_1_ distance of genes with a single transcript artificially decreases the average L_1_ distance.

### The MAQC data set

The RNA samples were sequenced on an Illumina GenomeAnalyzer resulting in 7 lanes per sample of 35 bp single-end reads [9]. The RNA-Seq data of the MAQC data set were downloaded from the NCBI read archive under accession number SRA010153, while the associated qPCR values were downloaded from the Gene Expression Omnibus (GEO) under accession number GSE5350. The reads of all 14 lanes were aligned to GRCh37/hg19 and Ensembl version 75 with Tophat2 [18]. Rather than the RefSeq annotation, Ensembl version 75 was used in the experiments, since the Ensembl annotation contains in many cases more transcripts per gene than RefSeq and therefore yields a more challenging and also larger test set. Since the MAQC data set records the association between qPCR probes and RefSeq annotations it was necessary to select only those qPCR probes mapping to a single RefSeq annotation, which, in turn, has a unique Ensembl equivalent. This resulted in a test set containing 798 transcripts with on average 8.6 transcripts per gene. It should be noted that the implementation of PennSeq in PERL has to be considered a proof-of-concept and as such fails to produce an output for around 10% of the test set.

Since the RNA-Seq data from MAQC are single-end the fragment length *l*(*r*) is unknown and was summed out of Eq (4). This sets the first term of the right-hand side of Eq (4) to 1. As the fragment start the down-stream end of each read was selected.

### The SEQC data set

We downloaded the SEQC data from the NCBI read archive under accession number GSE47792. On the BGI data we studied the ratio between the concentration of a single transcript for samples C and D and compared this to the expected ratio based on the concentration calculated for samples A and B. The latter is given as follows

$$\begin{array}{c} \frac{C}{D}=\frac{k_{1}A+\left(1-k_{1}\right)B}{k_{2}A+\left(1-k_{2}\right)B} \end{array}$$ (12)

where *k*_1_ = 3*z*/(3*z* + 1) and *k*_2_ = *z*/(*z* + 3) and, according to [25], *z* = 1.43. We evaluate on transcripts for which both sides of Eq (12) are well-defined for all methods. This gives us a test set of 76514 transcripts.

## Supporting information

S1 Appendix Further properties of Mix^2^. (PDF)

S2 Appendix Additional Tables and Figures. The tables in this appendix contain all the results from the experiments on the MAQC data.
(PDF)
