# Supplementary material for: Mixture models reveal multiple positional bias types in RNA-Seq data and lead to accurate transcript concentration estimates
Source: PLoS Comput Biol. 2017 May 15;13(5):e1005515. doi: 10.1371/journal.pcbi.1005515 (PMC5448817; doi:10.1371/journal.pcbi.1005515)
Supplement: S1 Appendix — (PDF) [file pcbi.1005515.s001.pdf]

S1 Appendix
Further properties of Mix^2

Andreas Tuerk, Gregor Wiktorin, Serhat Güler

Lexogen GmbH
Campus Vienna Biocenter 5, 1030 Vienna, Austria

November 29, 2016

1 Parameter estimation for the Mix^2 model

1.1 Derivation of the EM update formulas

The Expectation Maximization (EM) algorithm [1] increases the likelihood $L(R|\theta)$ of a data set R under a model $p(R|\theta)$ by maximizing, or more generally increasing, the auxiliary function

$$Q(\theta'|\theta) = E_{Z|R,\theta}(\log p(R, z|\theta')) \tag{1}$$

Here, θ is the current parameter set of the model $p(R|\theta)$ and θ' is the new parameter set that needs to be optimized. In addition, $Z = (z_r)_{r\in R}$ is a sequence of random hidden variables z_r and, hence, the expression on the right hand side of (1) is the expected value of $\log p(R, z|\theta')$, where z is one realization of Z, with respect to the random variable Z given R and θ. The hidden variables in the Mix^2 model are the transcript variable, $t = i$, and the mixture variable, $b = j$.

A necessary condition for the maximization of $Q(\theta'|\theta)$ is that the gradient of $Q(\theta'|\theta)$ equals zero, i.e.

$$\frac{\partial}{\partial\theta'} Q(\theta'|\theta) = 0 \tag{2}$$

For the Mix^2 model this means that

$$\frac{\partial}{\partial\alpha_i} Q(\theta'|\theta) = 0 \tag{3}$$

and

$$\frac{\partial}{\partial\beta_{kj}} Q(\theta'|\theta) = 0 \tag{4}$$

where i is the index of transcript $t = i$ and k is the index of group $g = k$. As usual, the update formula of the relative abundances α_i is given by

$$\alpha_i^{(n+1)} = \frac{1}{|R|}\sum_r p^{(n)}\left(t = i|r\right) \tag{5}$$

where $\alpha_i^{(n+1)}$ and $p^{(n)}(t = i|r)$ are the relative abundance and posterior probability after the $n+1$-th and n-th iteration of the EM algorithm. In addition to (4), the β_{kj} have to satisfy the constraint

$$\sum_{j=1}^{M} \beta_{kj} = 1 \tag{6}$$

where M is the number of mixture components. This constraint can be enforced with the Lagrange method. Taking the derivative with respect to β_{kj} leads to

$$\sum_{r\in R} p(g = k, b = j|r) + \beta_{kj}\lambda = 0 \tag{7}$$

which after some rearrangement results in

$$\beta_{kj}^{(n+1)} = \frac{\sum_r p^{(n)}\left(g = k, b = j|r\right)}{\sum_r p^{(n)}\left(g = k|r\right)} \tag{8}$$

where, as previously, $\beta_{kj}^{(n+1)}$ and $p^{(n)}(\cdot)$ are the mixture components and posterior probabilities after the $n+1$-th and after the n-th iteration, respectively. The posterior probabilities in (8) are given by

$$p^{(n)}(g = k, b = j|r) = \sum_{i\in k} p^{(n)}(t = i, b = j|r) \tag{9}$$

and

$$p^{(n)}(g = k|r) = \sum_{i\in k} p^{(n)}(t = i|r) \tag{10}$$

where the sums in (9) and (10) extend over all transcripts $t = i$ in group $g = k$ and the posteriors on the right-hand side of these equations can be derived according to Bayes formula as follows

$$p^{(n)}(t = i, b = j|r) = \frac{\alpha_i^{(n)}\beta_{ij}^{(n)} p(r|t = i, b = j)}{\sum_{ij}\alpha_i^{(n)}\beta_{ij}^{(n)} p(r|t = i, b = j)} \tag{11}$$

and

$$p^{(n)}(t=i|r) = \frac{\sum_j \alpha_i^{(n)} \beta_{ij}^{(n)} p(r|t=i,b=j)}{\sum_{ij} \alpha_i^{(n)} \beta_{ij}^{(n)} p(r|t=i,b=j)} \tag{12}$$

The posterior probability $p(r|t=i,b=j)$ in (11) and (12) is independent of the iteration. In the main paper the $p(r|t=i,b=j)$ where chosen to be Gaussians which are equidistantly distributed across the transcript $t=i$.

Without any tying, the group $g=k$ consists of a single transcript $t=i$ and (8) therefore becomes

$$\beta_{ij}^{(n+1)} = \frac{\sum_r p^{(n)}(t=i,b=j|r)}{\sum_r p^{(n)}(t=i|r)} \tag{13}$$

For global tying, on the other hand, the group consists of all the transcripts within the locus and therefore

$$p(g=k|r) = 1 \tag{14}$$

As a result, the update formula (8) becomes

$$\beta_j^{(n+1)} = \frac{1}{|R|} \sum_r p^{(n)}(b=j|r) \tag{15}$$

It is interesting to note, that (15) is similar to the update formula for the relative abundances α_i, equation (5). This is the case, because for global tying the following holds

$$p(r) = \sum_j \beta_j p(r|b=j) \tag{16}$$

which is similar to the superposition

$$p(r) = \sum_i \alpha_i p(r|t=i) \tag{17}$$

Multi-mapping reads and sequence specific bias

The previous discussion assumes that a fragment r maps uniquely to the genomic reference. If, on the other hand, fragment r has multiple hits $H(r)$ on the reference, then

$$p(h|r) = \frac{p(h)}{\sum_{h \in H(r)} p(h)} \tag{18}$$

needs to be taken into account when estimating the parameters of the Mix^2 model. Rather than calculating (18) during parameter estimation $p(h|r)$ is often set to $1/\#H(r)$ [2]. Equation (18) can be extended to cover the situation of a sequence specific bias. In this case, the probability that a sequence $seq(r)$ within or surrounding fragment r is generated can be smaller than 1 and the right-hand side of equation (18) needs to be multiplied by this sequence specific probability, $p(generate|seq(r))$. The probability $p(generate|seq(r))$ can, for instance, be estimated as in [4] by calculating the ratio of the probability of the sequence $seq(r)$ under the biased model to the uniform model. Most commonly, $seq(r)$ is a sequence directly preceding or following r and $p(generate|seq(r))$ therefore reflects the probability that a primer with start sequence $seq(r)$ anneals to the sample. Details on how equation (18) and its generalization to a sequence specific bias fits into the parameter estimation of the Mix^2 model are given in Section "Parameter estimation". It should be noted that in our current implementation of the Mix^2 model we do not take sequence specific bias into consideration, nor do we use (18) to calculate the posterior probability of a hit.

If fragment r has multiple hits $H(r)$ and a sequence specific bias then

$$p(t=i,b=j|r) = \sum_{h \in H(r)} p(t=i,b=j|h)p(h|r) \tag{19}$$

and the update formula for β_{kj}, equation (8), becomes

$$\beta_{kj}^{(n+1)} = \frac{\sum_{r \in R} \sum_{h \in H(r)} p^{(n)}(g=k,b=j|h)p(h|r)}{\sum_{r \in R} \sum_{h \in H(r)} p^{(n)}(g=k|h)p(h|r)}. \tag{20}$$

Here $p(h|r)$ is given by equation (18) or the right-hand side of equation (18) multiplied by $p(generate|seq(r))$ the probability of generating the sequence $seq(r)$, which is either part of or surrounding fragment r.

1.2 Identifiability and uniqueness of maximum likelihood solution

The Mix^2 model is identifiable on the set of fragments R iff the mapping $\theta \to p_\theta(R)$ is injective, where, as in the previous section, θ is the vector of pairs of parameters

$$\theta = ((a_i, b_{i,j}))_{i=1,\ldots,N \wedge j=1,\ldots,M} \tag{21}$$

The mapping $\theta \to p_\theta(R)$ is given by the product of two mappings

$$p_\theta(R) = A \cdot M \cdot \theta \tag{22}$$

where A is the linear map given by

$$A = (a_{r,(i,j)})_{r \in R \wedge (i,j) \in (1,\ldots,N) \times (1\ldots,M)} \tag{23}$$

with

$$a_{r,(i,j)} = p(r|t=i, b=j) \tag{24}$$

which is the value of the j-th Gaussian of transcript i for fragment r. Hence r is an index for the rows and the pair (i,j) is an index for the columns of A. The second mapping in (22) is componentwise multiplication of θ given by

$$M(\theta) \to ((a_i b_{i,j}))_{i=1,\ldots,N \wedge j=1,\ldots,M} \tag{25}$$

The mapping M is invertible on the parameters θ since

$$\sum_j \alpha_i \beta_{ij} = \alpha_i \tag{26}$$

and thus equation (22) is injective iff A is injective on the set $M\theta$, which is the $NM-1$ simplex Δ^{NM-1}. This condition can be checked by first checking the stronger condition of injectivity of A on the full linear space $\mathbb{R}^{N\times M}$. If A is injective on $\mathbb{R}^{N\times M}$ then, clearly, A is injective on Δ^{NM-1}. If, on the other hand, A is not injective on $\mathbb{R}^{N\times M}$ then it is necessary to check whether differences of elements in Δ^{NM-1} other than 0 lie in the kernel of A on $\mathbb{R}^{N\times M}$. The latter will be the case if the dimension of the kernel of A is greater than 1, since then

$$dim\,(ker(A)) + dim\left(\Delta^{NM-1}\right) > dim\left(\mathbb{R}^{N\times M}\right) \tag{27}$$

The dimension of the kernel of A is, for instance, greater than 1 if two transcripts $t=i$ and $t=i'$ share the same Gaussian $b=j$ and $b=j'$, which happens only if the transcripts have the same length and their exons are properly aligned. This situation can be avoided by shifting the Gaussians $p(r|t=i,b=j)$, $p(r|t=i',b=j')$ away from each other, which ensures that

$$p(r|t=i, b=j) \neq p(r|t=i', b=j') \tag{28}$$

and removes therefore identical columns in A. Shifting the Gaussians means that some of them are not equidistantly distributed along a transcript but has otherwise a minor effect on the properties of the Mix^2 model. Summarizing, we state the following

Proposition 1. *A sufficient condition for the identifiability of the Mix^2 model is the injectivity on $\mathbb{R}^{N\times M}$ of the matrix A in equations (23) and (24). If the Mix^2 model fails to be identifiable because two transcripts $t=i$ and $t=i'$ share one Gaussian for two of their mixture components $b=j$ and $b=j'$, then the Mix^2 model can be made identifiable by shifting the Gaussians $p(r|t=i,b=j)$, $p(r|t=i',b=j')$ away from each other.*

Equation (26) shows further that the Mix^2 model is equivalent to a mixture model of the distributions $p(r|t=i,b=j)$ with mixture weights c_{ij} if no Gaussian is shared between two transcripts. In this case, the maximum likelihood solution for the c_{ij} is unique, since the log likelihood surface of mixture models is concave [3], and the c_{ij} and the parameters of the Mix^2 model stand in a one-to-one relationship. This can be summarized as follows.

Proposition 2. *The Mix^2 model is equivalent to a mixture of the distributions $p(r|t=i,b=j)$ with respective mixture weights c_{ij} if no two transcripts share the same Gaussian. Since the log likelihood function for a mixture*

is concave there exists a unique maximum likelihood solution for the c_{ij} to which the EM algorithm converges. The α_i and β_{ij} of the Mix^2 model can be derived, in this case, from the c_{ij} as follows.

$$\alpha_i = \sum_{j=1}^{M} c_{ij} \tag{29}$$

$$\beta_{ij} = \frac{c_{ij}}{\alpha_i} \tag{30}$$

2 Fragment start distributions in Cufflinks

The Mix^2 model in the main paper factorizes the transcript specific fragment distribution $p(r|t=i)$ as follows

$$p(r|t=i) = p(s(r)|t=i)p(l(r)|s(r), t=i) \tag{31}$$

where $s(r)$ and $l(r)$ are the start and length of fragment r. Cufflinks [5], on the other hand, reverses the order of $s(r)$ and $l(r)$ in (31) and factorizes $p(r|t=i)$ according to

$$p(r|t=i) = p(l(r)|t=i)p(s(r)|l(r), t=i) \tag{32}$$

The fragment length distribution $p(l(r)|t=i)$ in (32) is derived from the cumulative distribution of fragment lengths $p(l(r))$ for the complete data set. For this purpose, $p(l(r))$ is truncated to the possible fragment lengths for transcript $t=i$ and subsequently renormalized such that

$$\sum_{l=1}^{l(t=i)} p(l|t=i) = 1 \tag{33}$$

where $l(t=i)$ is the length of transcript $t=i$. The fragment start distribution $p(s(r)|l(r), t=i)$, on the other hand, is assumed to be uniform over the possible fragment starts $s(r)$ for transcript $t=i$ and fragment length $l(r)$, i.e.

$$p(s(r)|l(r), t=i) = \frac{1}{l(t=i) - l(r) + 1} \tag{34}$$

The fragment start distribution $p(s(r)|t=i)$ for $t=i$ according to the Cufflinks model can be derived by summing $l(r)$ out of (32). In the absence of fragment length information, e.g. for single-end RNA-Seq data, Cufflinks assumes by default a Gaussian with mean 200 and standard deviation 80 for the cumulative fragment length distribution $p(l(r))$. For this default setting the fragment start distribution $p(s(r)|t=i)$ is given in Figure 2 (a) of the main article for transcripts with length between 400 bps and 3000 bps. It can be seen that for long transcripts the Gaussian distribution $p(l(r))$ produces a short and steep tail at the end of $p(s(r)|t=i)$, whereas this tail shifts increasingly to the 5' end of the transcript for shorter transcripts. The assumption of a Gaussian with mean 200 and standard deviation 80 corresponds to a size selection of the fragments prior to sequencing. Thus, Figure 2 (a) in the main text shows that even for a uniform fragment distribution, size selection generates a transcript length specific bias.

References

[1] A. P. Dempster, N. M. Laird, and D. B. Rubin. Maximum likelihood from incomplete data via the EM algorithm. *Journal of the Royal Statistical Society, Series B*, 39(1):1–38, 1977.

[2] Ali Mortazavi, Brian A Williams, Kenneth McCue, Lorian Schaeffer, and Barbara Wold. Mapping and quantifying mammalian transcriptomes by RNA-Seq. *Nature Methods*, 5(7):621–628, Jul 2008.

[3] Pachter and Sturmfels, editors. *Algebraic Statistics for Computational Biology.* Cambridge University Press, 2005.

[4] Adam Roberts, Cole Trapnell, Julie Donaghey, John L Rinn, and Lior Pachter. Improving RNA-Seq expression estimates by correcting for fragment bias. *Genome Biology*, 12(3):R22, Mar 2011.

[5] Cole Trapnell, Brian A Williams, Geo Pertea, Ali Mortazavi, Gordon Kwan, Marijke J van Baren, Steven L Salzberg, Barbara J Wold, and Lior Pachter. Transcript assembly and quantification by RNA-Seq reveals unannotated transcripts and isoform switching during cell differentiation. *Nature Biotechnology*, 28(5):511–515, May 2010.
