# Supplementary material for: Mixture models reveal multiple positional bias types in RNA-Seq data and lead to accurate transcript concentration estimates
Source: PLoS Comput Biol. 2017 May 15;13(5):e1005515. doi: 10.1371/journal.pcbi.1005515 (PMC5448817; doi:10.1371/journal.pcbi.1005515)
Supplement: S2 Appendix — The tables in this appendix contain all the results from the experiments on the MAQC data. (PDF) [file pcbi.1005515.s002.pdf]

S2 Appendix
Additional Tables and Figures

Andreas Tuerk, Gregor Wiktorin, Serhat Güler

Lexogen GmbH
Campus Vienna Biocenter 5, 1030 Vienna, Austria

April 26, 2017

gene	STR	TR	LT	ST	ES	A5DS	A3AS	IR
DAPK3	-	7	2257	599			×	×
HAUS5	+	10	4462	526	×	×	×	×
KLK5	-	5	1563	672	×		×	×
LDHD	-	4	2067	701		×	×	×
LGALS17A	+	6	2584	406	×		×	×
TESK2	-	7	3074	428	×			
USF2	+	15	2742	446	×		×	×

Table A: The 7 test genes from the Ensembl reference GRCh37 v75 and their variants of differential splicing. The first four columns contain the strand (STR) of the gene, the number of transcripts (TR) and the number of base pairs of the longest (LT) and shortest transcript (ST). The remaining columns indicate which types of differential splicing are present, i.e. exon skipping (ES), alternative 5' donor sites (A5DS), alternative 3' acceptor sites (A3AS) intron retention (IR).

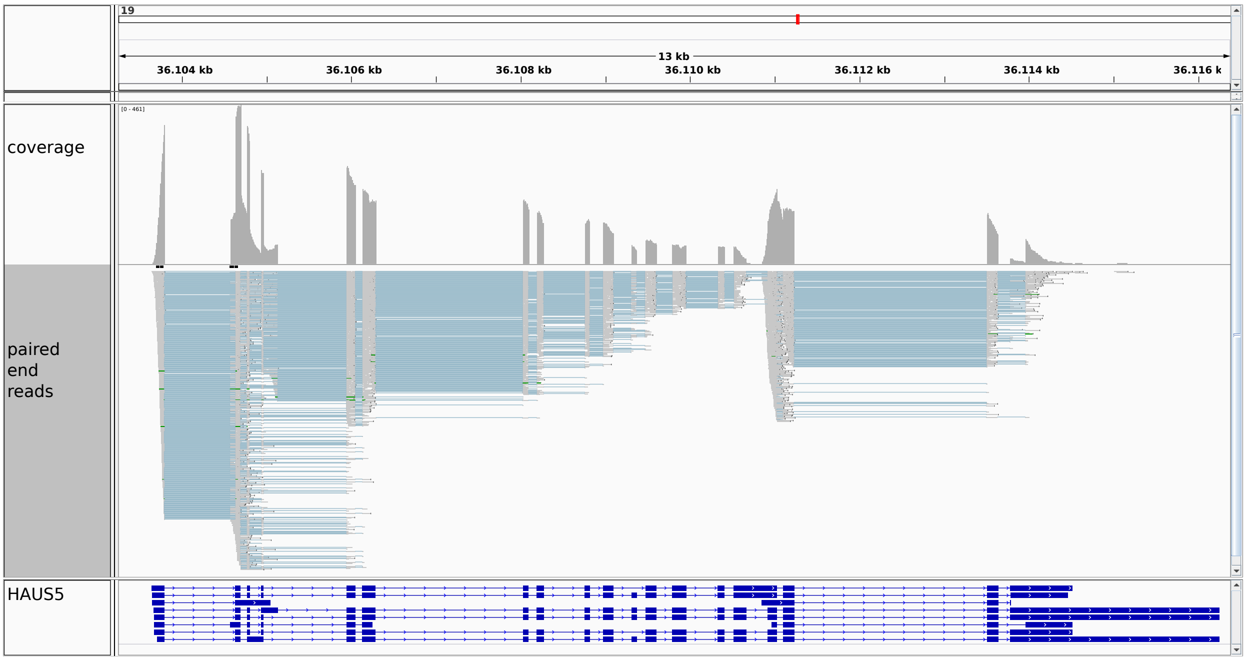

Figure A: Coverage and distribution of 1000 paired-end reads sampled from the HAUS5 gene with a 5' bias. This figure shows a snapshot of the IGV browser. The top track shows the coverage of the data, while the middle track shows the individual paired end reads. The bottom track shows the Ensembl annotation of the HAUS5 gene.

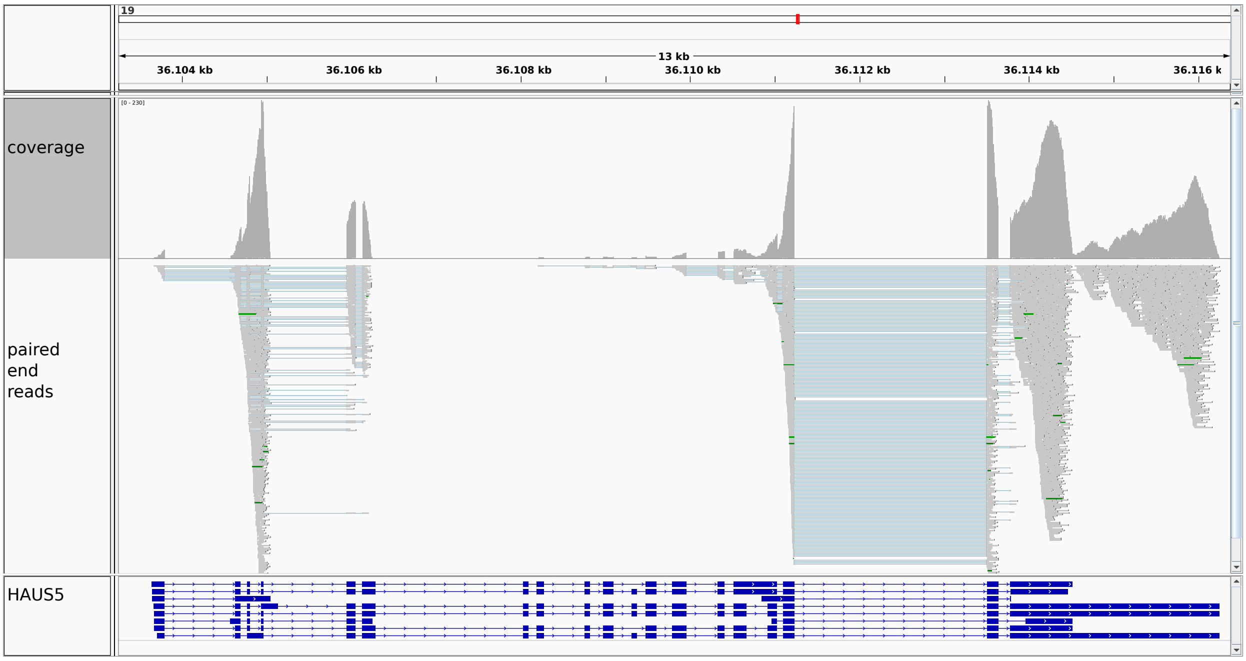

Figure B: Coverage and distribution of 1000 paired-end reads sampled from the HAUS5 gene with a 3' bias. This figure shows a snapshot of the IGV browser. The top track shows the coverage of the data, while the middle track shows the individual paired end reads. The bottom track shows the Ensembl annotation of the HAUS5 gene.

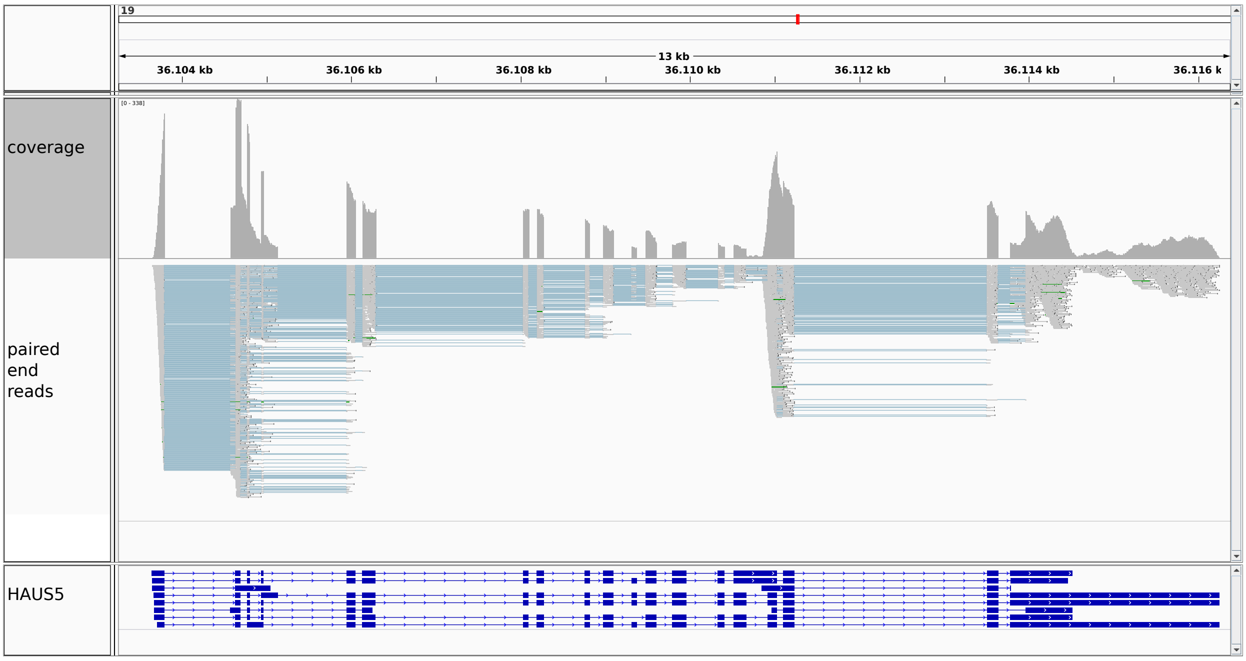

Figure C: Coverage and distribution of 1000 paired-end reads sampled from the HAUS5 gene with a 5'+3' bias. This figure shows a snapshot of the IGV browser. The top track shows the coverage of the data, while the middle track shows the individual paired end reads. The bottom track shows the Ensembl annotation of the HAUS5 gene.

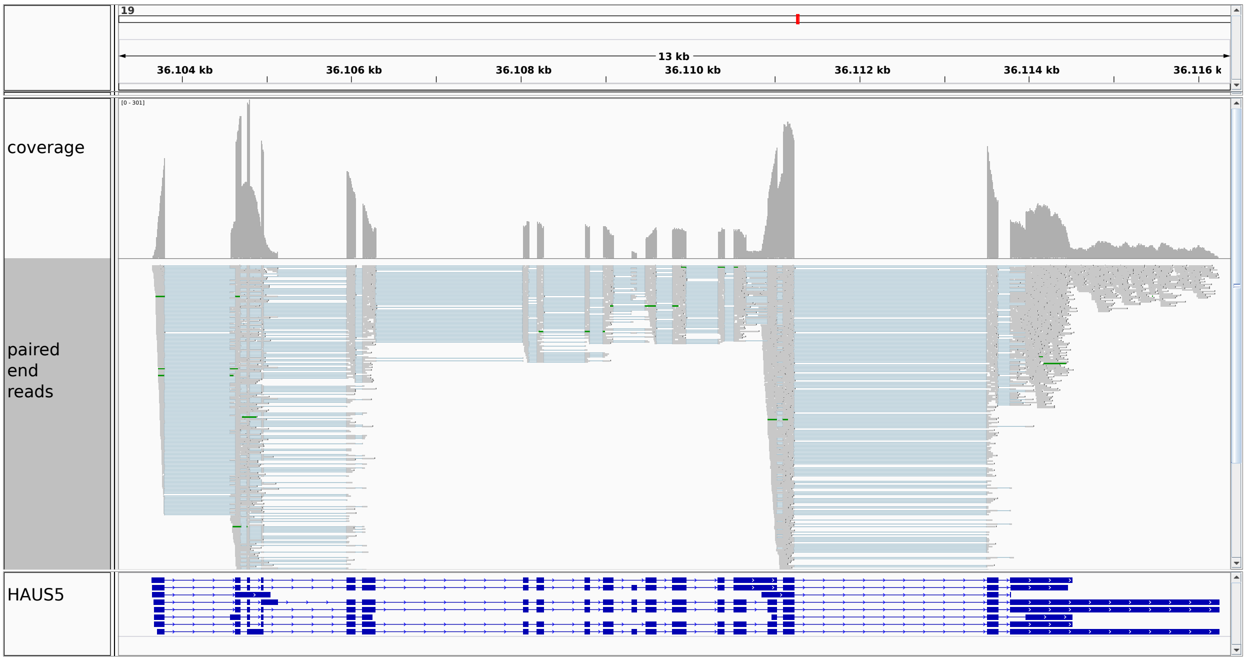

Figure D: Coverage and distribution of 1000 paired-end reads sampled from the HAUS5 gene with a Cufflinks bias. This figure shows a snapshot of the IGV browser. The top track shows the coverage of the data, while the middle track shows the individual paired end reads. The bottom track shows the Ensembl annotation of the HAUS5 gene.

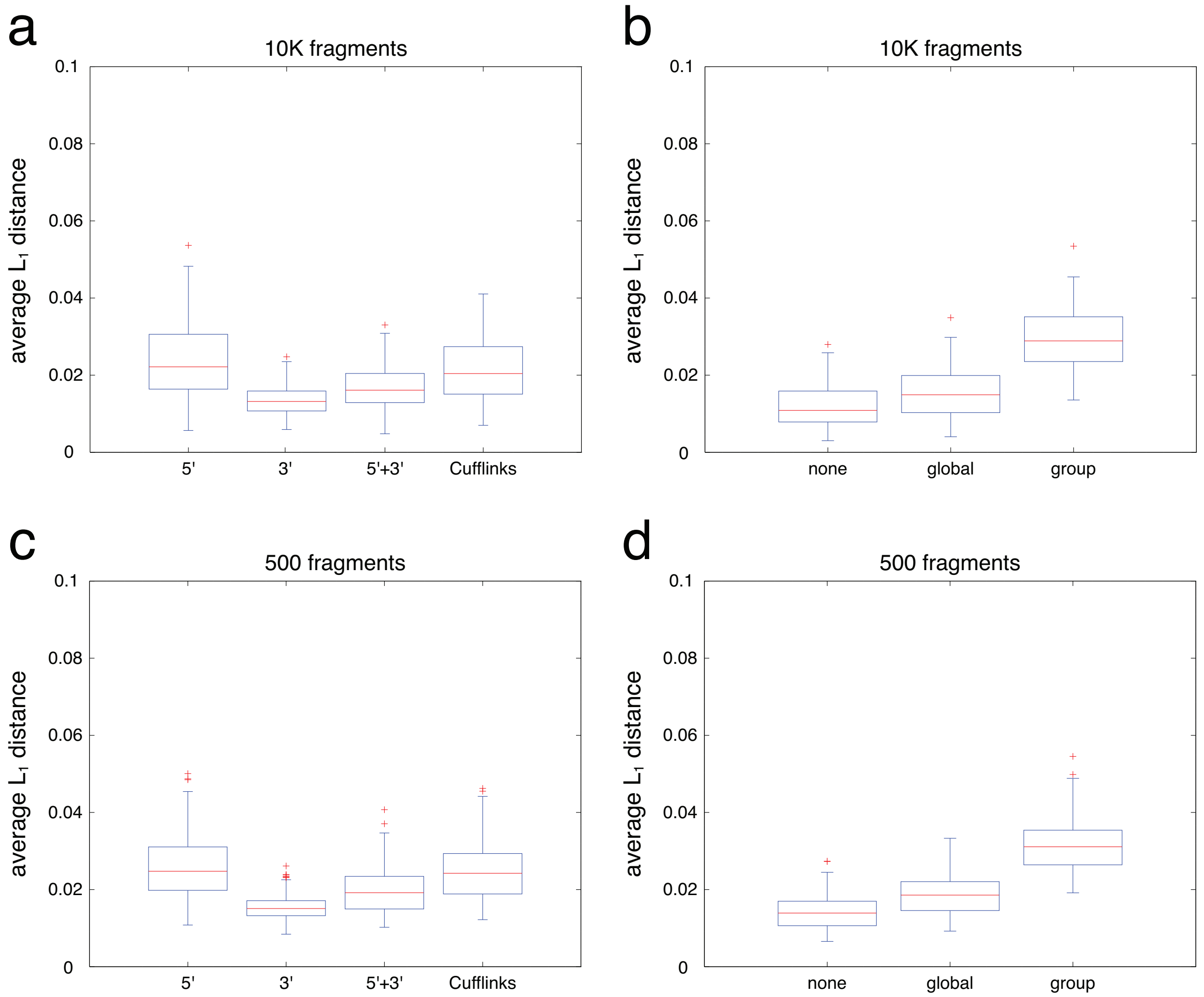

Figure E: Comparison between C++ and Octave versions of the Mix^2 software. (a), (b) Boxplots of L_1 distance between results for 10000 fragments. (c), (d) Boxplots of L_1 distance between results for 500 fragments. (a), (c) L_1 distance for bias types. (b), (d) L_1 distance for types of parameter tying.

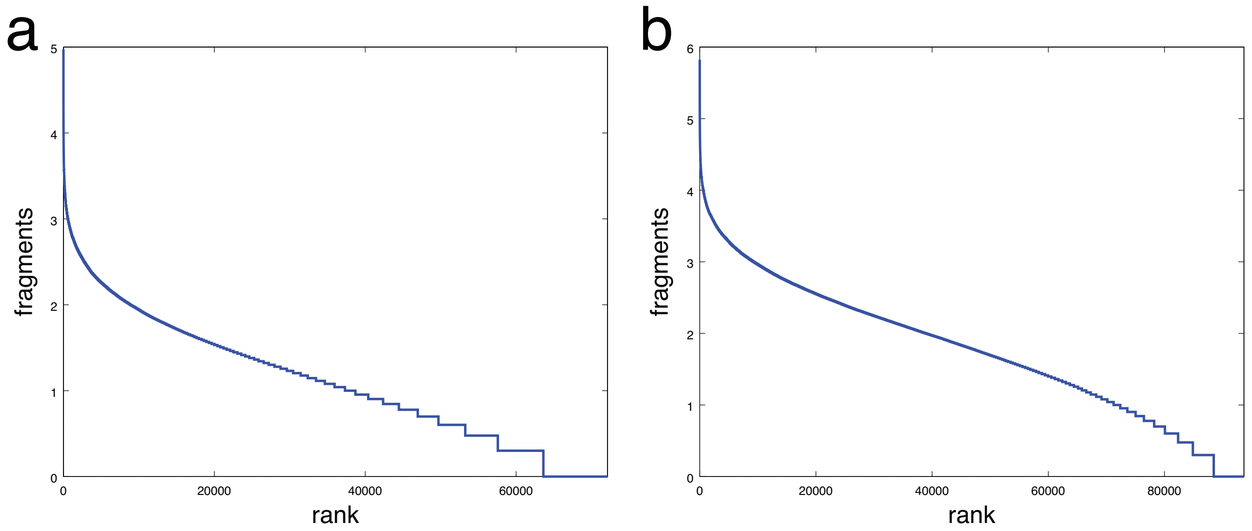

Figure F: Transcript expression profile of artificial data. Ranking of fragments per transcript for a total of 5 mio (a) and 50 mio fragments.

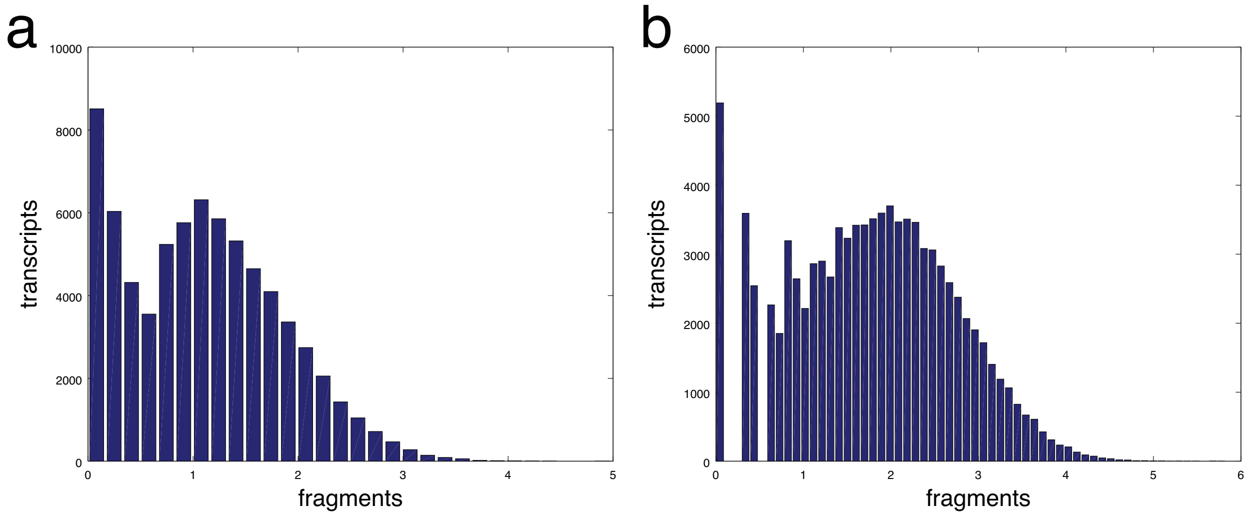

Figure G: Transcript expression profile of artificial data. Distribution of fragments per transcripts for a total of 5 mio (a) and 50 mio fragments.

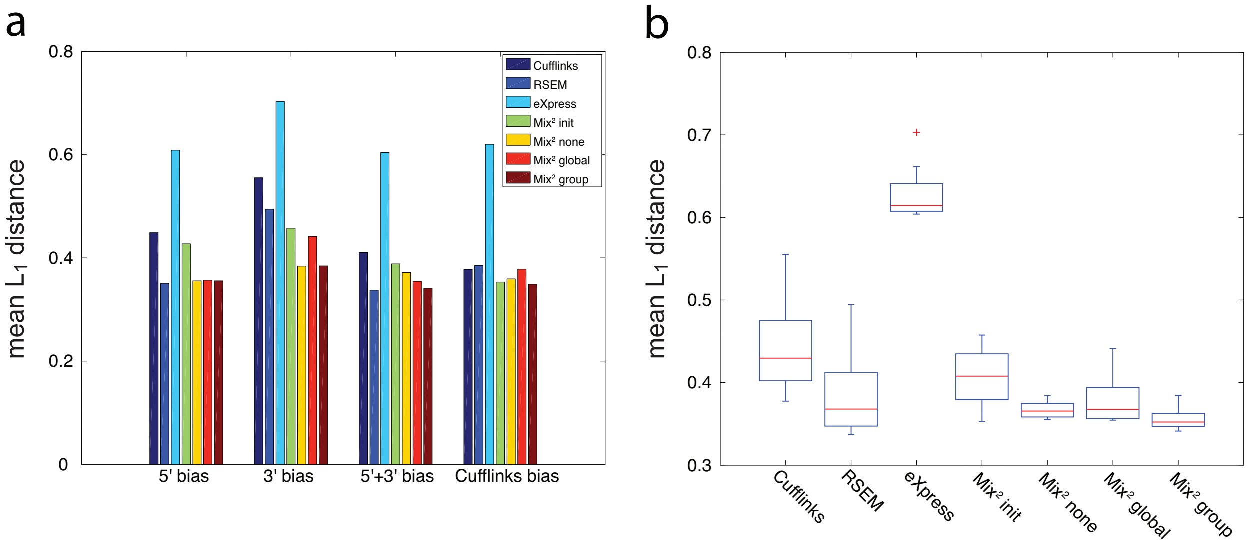

Figure H: Mean L_1 distance between estimated and correct relative abundances for Cufflinks, RSEM, eXpress and Mix^2 for 4 biases on full transcriptome and a total of 5 mio read pairs. Experiments also include results for Mix^2 with not adapted initial mixture weights β_{ij} (Mix^2 init). (b) boxplots of mean L_1 distances in (a) for all methods.

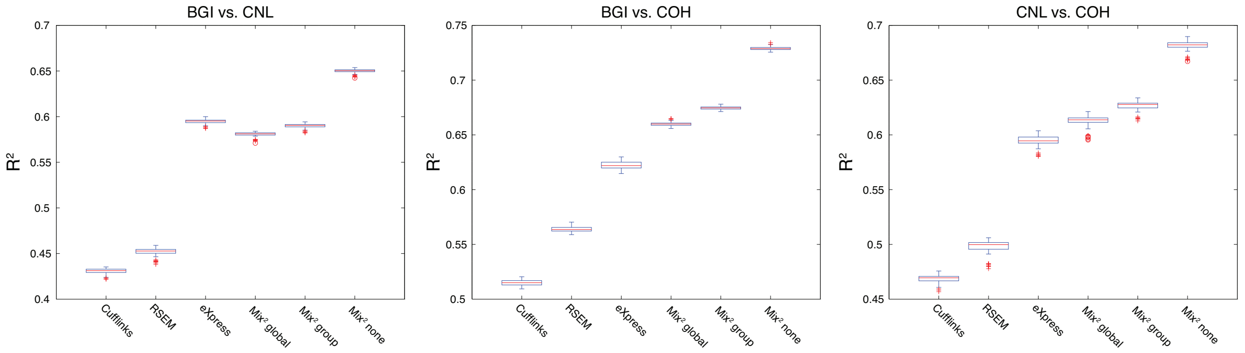

Figure I: R^2 values for pair-wise comparisons of sites BGI, CNL and COH in SEQC data.

AGR A	BGI A	BGI B	BGI C	BGI D	CNL A	COH A
49121	38240	39767	39493	40640	37679	53634

Table B: **Number of clustered fragment distributions for sites and samples in SEQC data.**

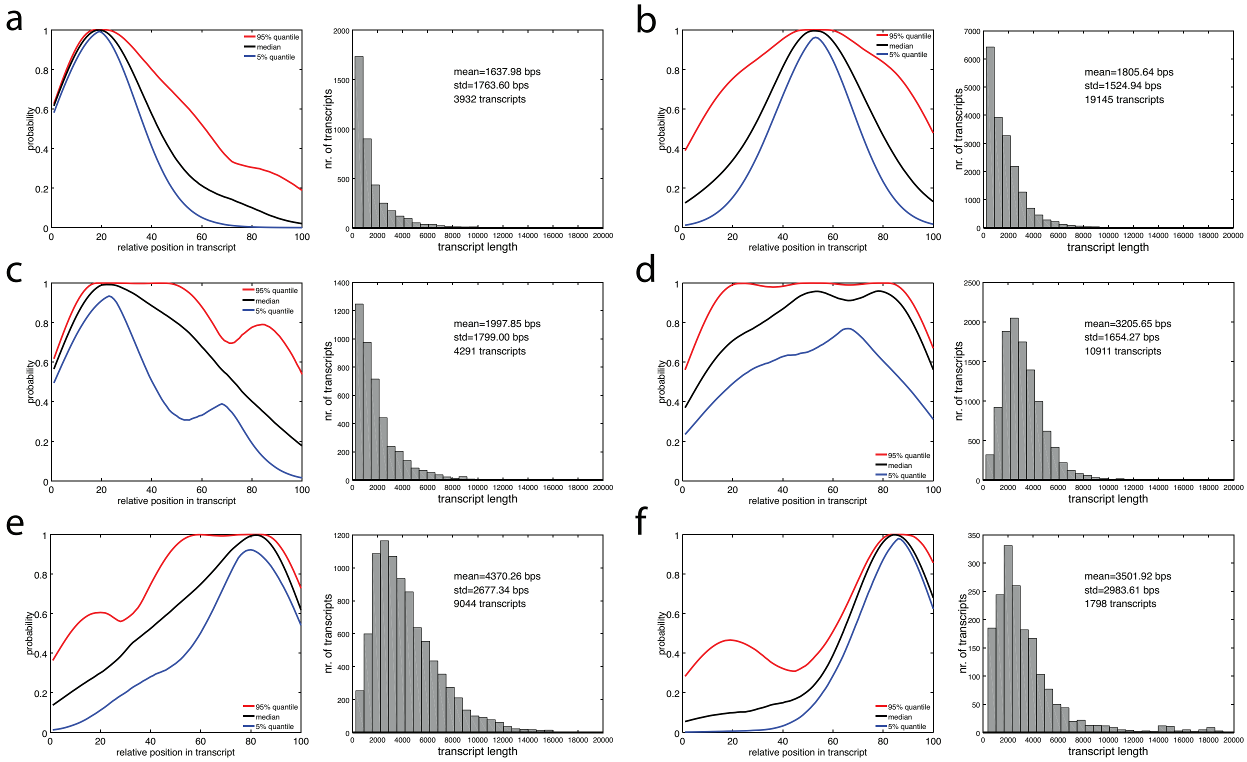

Figure J: Clusters for site AGR, sample A and flow-cell AC0C1TACXX in SEQC data.

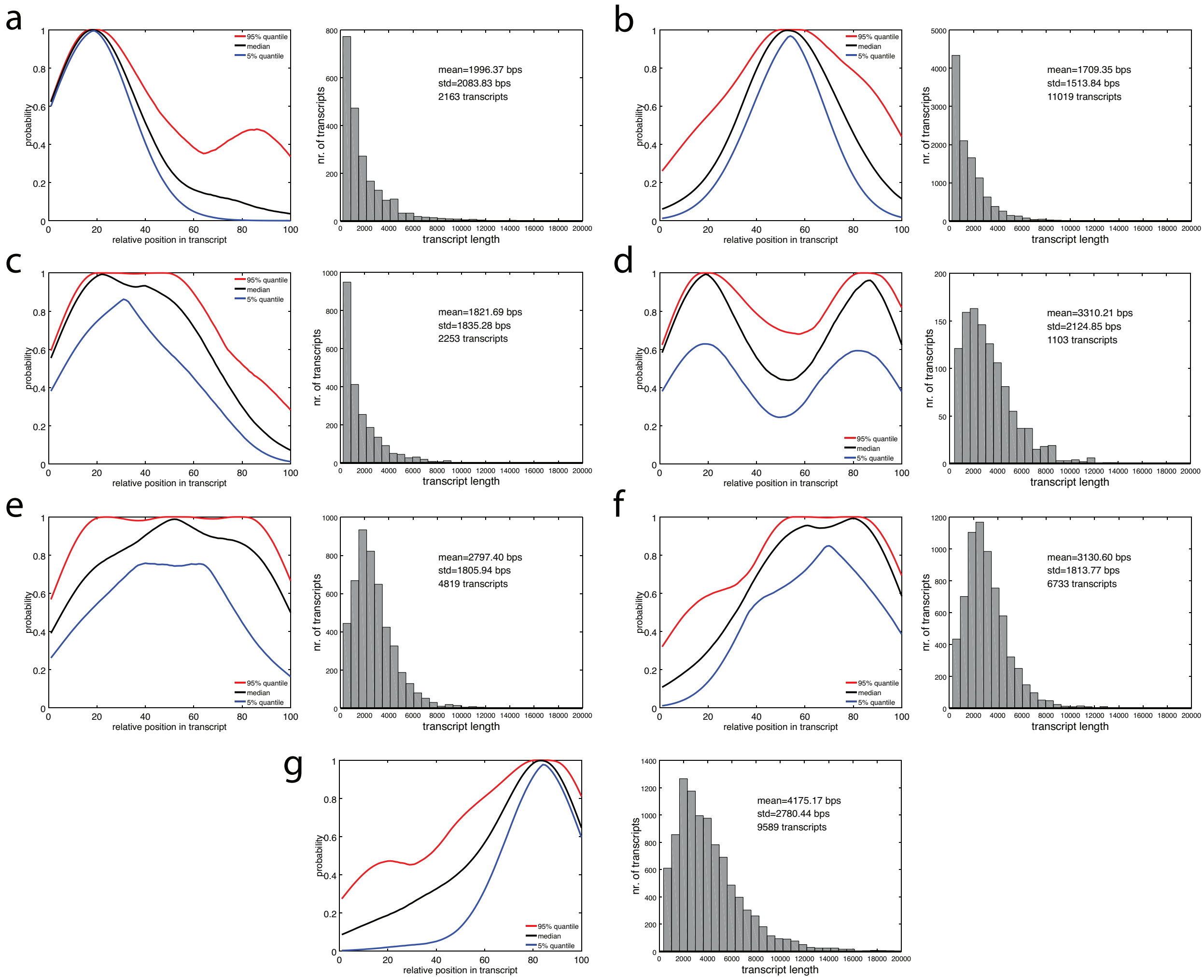

Figure K: Clusters for site CNL, sample A and flow-cell AD0FD7ACXX in SEQC data.

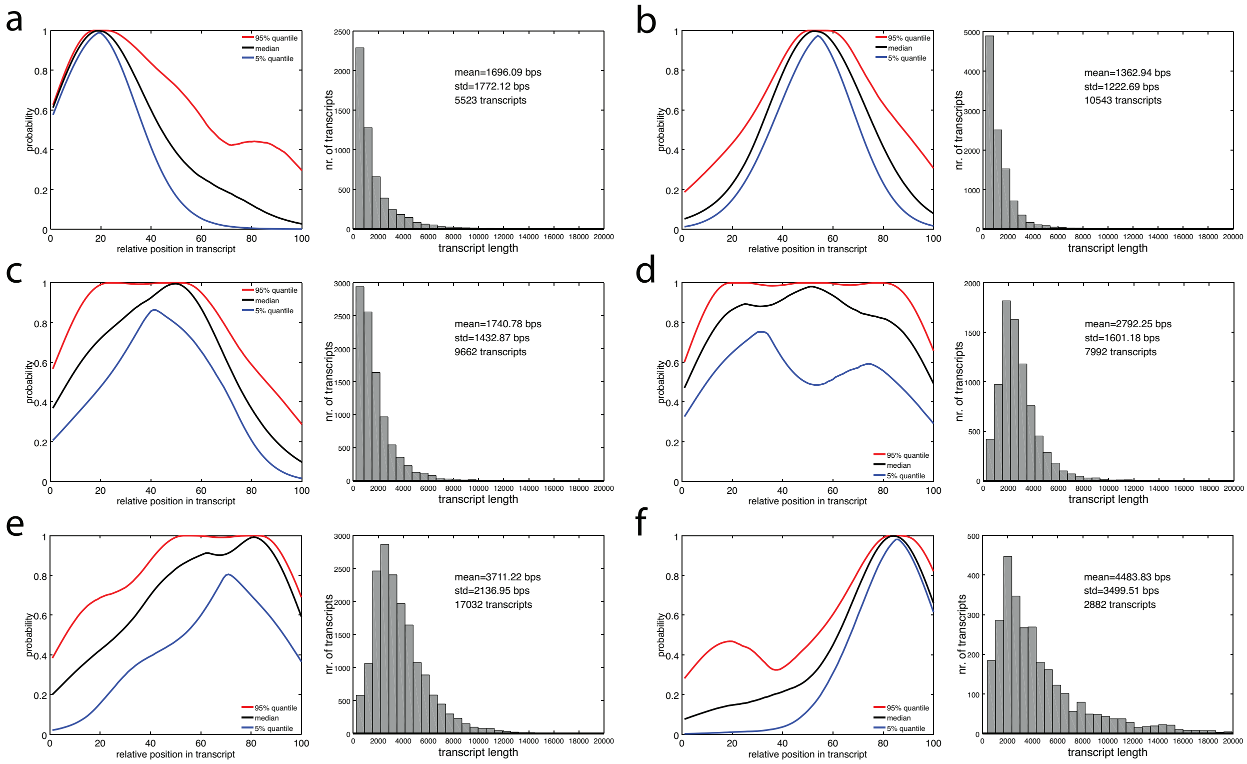

Figure L: Clusters for site COH, sample A and flow-cell AC03KTACXX in SEQC data.

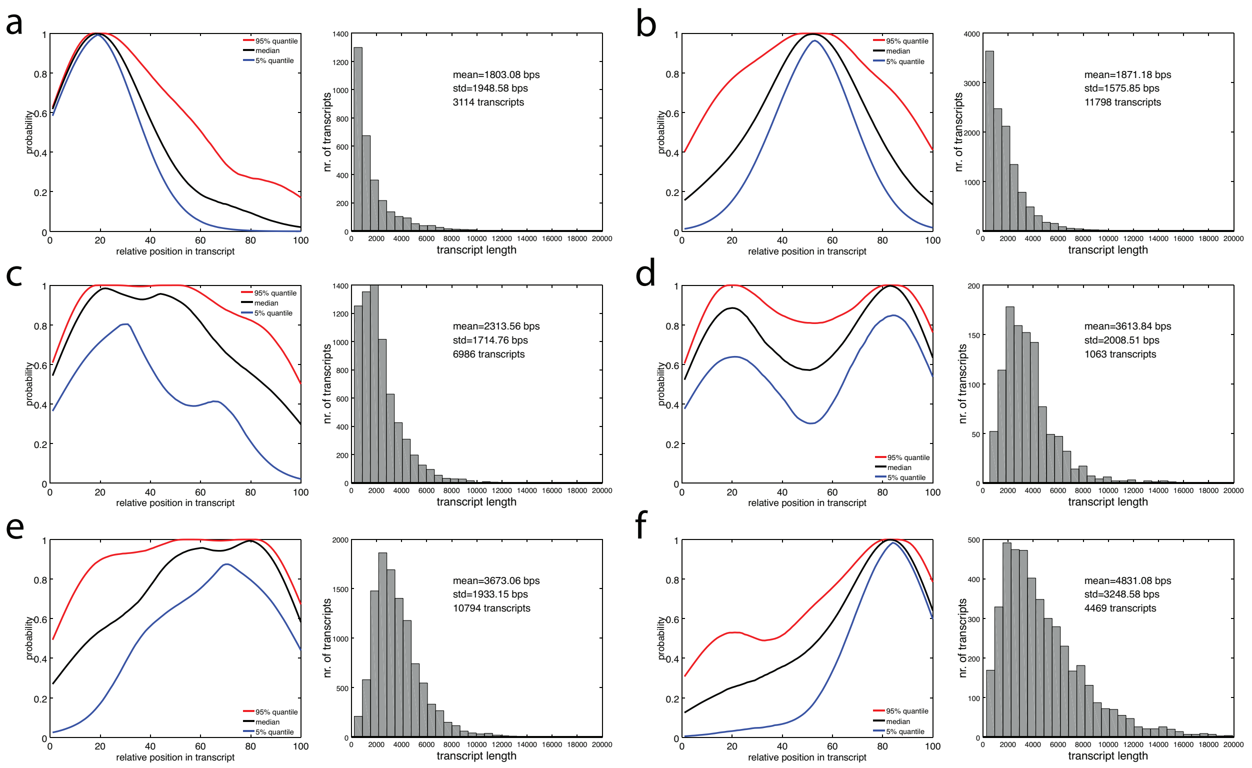

Figure M: Clusters for site BGI, sample A and flow-cell AC0AYTACXX in SEQC data.

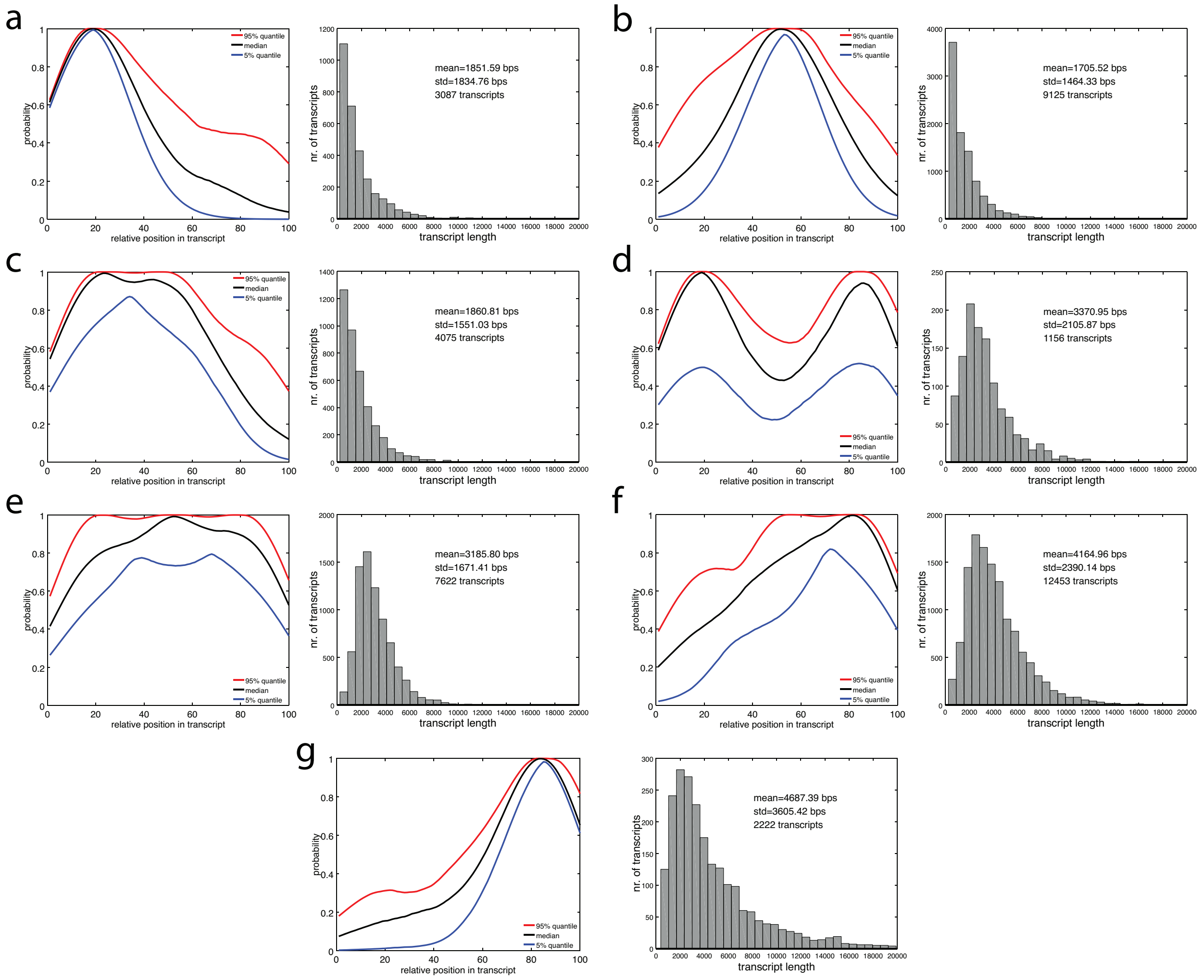

Figure N: Clusters for site BGI, sample B and flow-cell AC0AYTACXX in SEQC data.

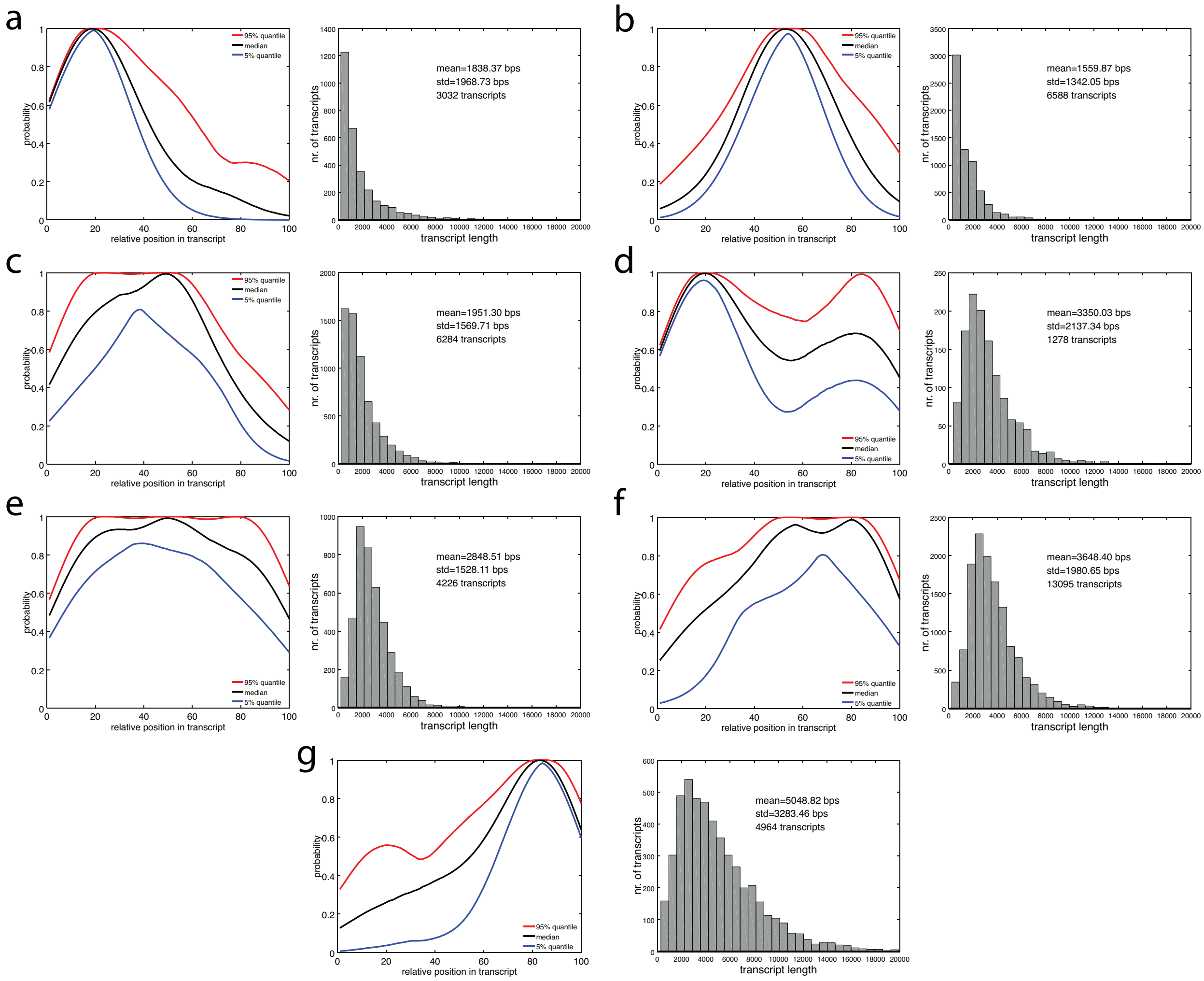

Figure O: Clusters for site BGI, sample C and flow-cell AC0AYTACXX in SEQC data.

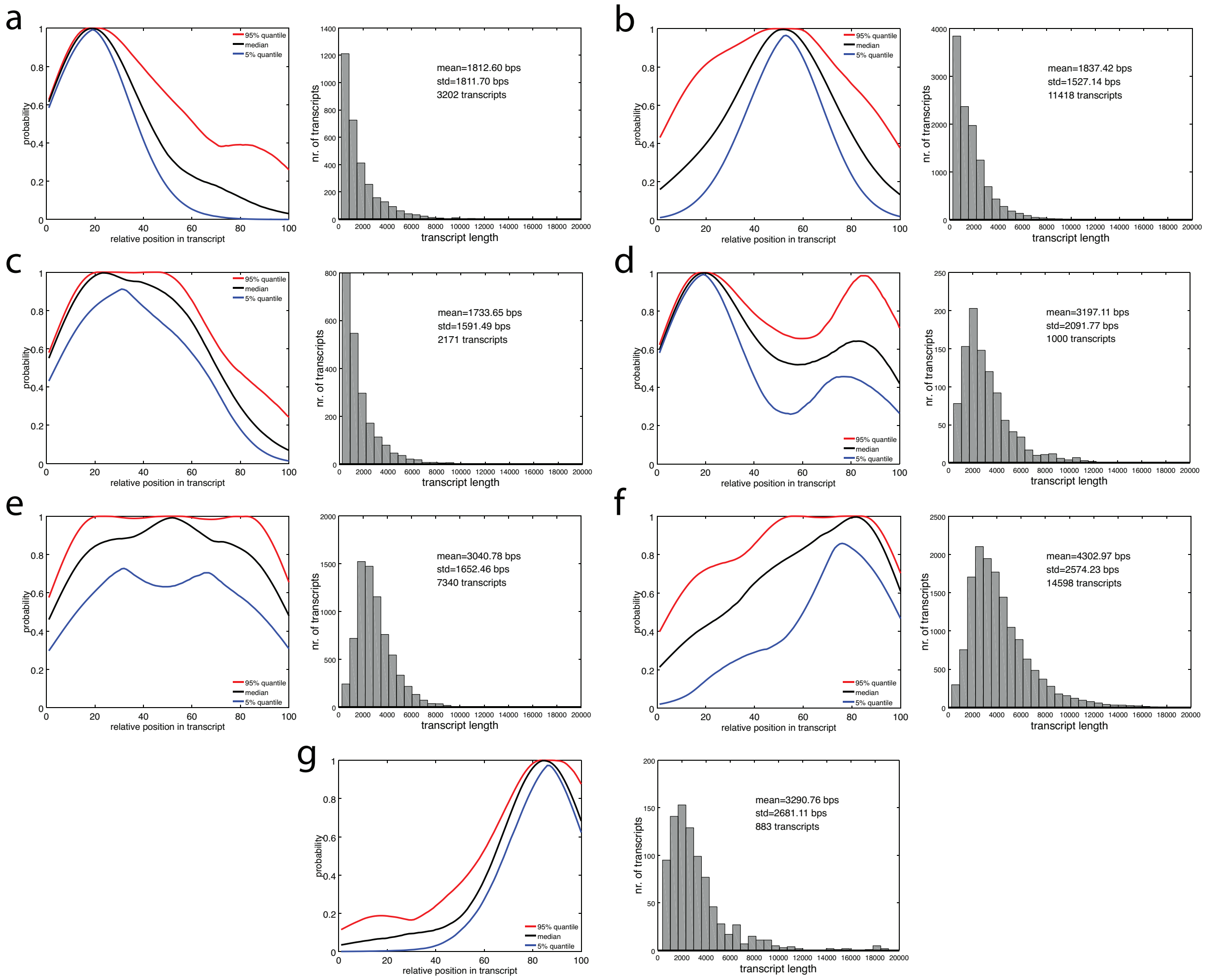

Figure P: Clusters for site BGI, sample D and flow-cell AC0AYTACXX in SEQC data.
